# *PODNL1* Methylation Serves as a Prognostic Biomarker and Associates with Immune Cell Infiltration and Immune Checkpoint Blockade Response in Lower-Grade Glioma

**DOI:** 10.3390/ijms222212572

**Published:** 2021-11-22

**Authors:** Humaira Noor, Ashraf Zaman, Charles Teo, Michael E. Sughrue

**Affiliations:** 1 Cure Brain Cancer Biomarkers and Translational Research Group, Prince of Wales Clinical School, University of New South Wales, Sydney, NSW 2031, Australia; 2 Adult Cancer Program, Lowy Cancer Research Centre, UNSW Sydney, Randwick, NSW 2031, Australia; 3 Faculty of Medicine, University of New South Wales, Randwick, NSW 2031, Australia; a.zaman@garvan.org.au; 4 Garvan-Weizmann Centre for Cellular Genomics, Garvan Institute of Medical Research, Darlinghurst, Sydney, NSW 2010, Australia; 5 Centre for Minimally Invasive Neurosurgery, Prince of Wales Private Hospital, Randwick, NSW 2031, Australia; charlie@neuroendoscopy.info (C.T.); michael.sughrue@o8t.com (M.E.S.)

**Keywords:** *Podocan-like 1*, *PODNL1*, glioma, low-grade glioma, CpG methylation, G-CIMP, immune infiltration, immune checkpoint blockade

## Abstract

Lower-grade glioma (LGG) is a diffuse infiltrative tumor of the central nervous system, which lacks targeted therapy. We investigated the role of *Podocan-like 1* (*PODNL1*) methylation in LGG clinical outcomes using the TCGA-LGG transcriptomics dataset. We identified four *PODNL1* CpG sites, cg07425555, cg26969888, cg18547299, and cg24354933, which were associated with unfavorable overall survival (OS) and disease-free survival (DFS) in univariate and multivariate analysis after adjusting for age, gender, tumor-grade, and *IDH1*-mutation. In multivariate analysis, the OS and DFS hazard ratios ranged from 0.44 to 0.58 (*p* < 0.001) and 0.62 to 0.72 (*p* < 0.001), respectively, for the four *PODNL1* CpGs. Enrichment analysis of differential gene and protein expression and analysis of 24 infiltrating immune cell types showed significantly increased infiltration in LGGs and its histological subtypes with low-methylation levels of the *PODNL1* CpGs. High *PODNL1* expression and low-methylation subgroups of the *PODNL1* CpG sites were associated with significantly increased *PD-L1, PD-1*, and *CTLA4* expressions. *PODNL1* methylation may thus be a potential indicator of immune checkpoint blockade response, and serve as a biomarker for determining prognosis and immune subtypes in LGG.

## 1. Introduction

Lower-grade gliomas (LGGs) are diffusely infiltrative tumors of the central nervous system (CNS), which consist of WHO grades II and III astrocytomas and oligodendrogliomas [1,2]. Adult LGGs typically affect young patients with a mean age of 41 [3], and are ultimately fatal with a median overall survival between 4 and 13 years [4]. Over the past 30 years, there has been no significant improvement in LGG clinical outcome [3], and with increasing recent knowledge of distinct molecular subgroups within LGGs and its histological subtypes [5,6,7,8], there is a need to identify therapeutically targetable drivers of tumor aggressiveness and malignant transformation. Currently, ongoing clinical trials are underway for targeting the well-established diagnostic and prognostic biomarker *isocitrate dehydrogenase 1/2* (*IDH1*/*2*) mutation [9,10,11]. There is no molecular targeted therapy under development for the more aggressive *IDH1*/*2* wildtype LGGs [11,12], or the aggressive subgroup within *IDH1*/*2* mutant LGGs [13,14]. Thus, there is a need to identify biomarkers associated with LGG aggressiveness that may serve as therapeutic targets and facilitate the discovery of efficacious treatment options for LGG.

*Podocan Like 1* (*PODNL1*) is a member of the small leucine-rich proteoglycan (SLRP) family [15], and it belongs to the Class V non-canonical class of SLRPs in particular [16,17]. It is highly expressed in tissues and bones [15] and high-grade gliomas [18]. A recent study reported the unfavorable effects of *PODNL1* overexpression in glioma overall survival [19]. It has also been reported as a prognostic marker in ovarian cancer [20]. However, the mechanisms of these effects or the *PODNL1* regulation machinery remained unknown, as the exploration and characterization of this gene are still in its dawn.

As the frequent *IDH1*/*2* mutation induces global DNA hypermethylation, commonly known as glioma CpG island methylator phenotype (G-CIMP) in LGG [6], in this study, we first investigated the correlation between *PODNL1* methylation and *PODNL1* mRNA expression in TCGA-LGG to determine whether *PODNL1* expression is epigenetically regulated, and identified specific methylated CpG islands that significantly correlate with *PODNL1* mRNA expression. We next determined the prognostic effects of *PODNL1* mRNA overexpression in LGG disease-free survival (DFS) in order to elucidate its potential role in affecting LGG aggressiveness. We also performed comprehensive overall and disease-free univariate and multivariate survival analysis based on individual *PODNL1* CpG site methylations to identify significantly prognostic *PODNL1* CpGs. Lastly, we performed differential gene and protein expression analysis based on the methylation levels of all significant CpGs individually, in order to shed light on the mechanism involved in driving LGG aggressiveness, which revealed significant differences in tumor immune microenvironment, thus we further investigated the potential response to immune checkpoint blockade therapies based on methylation subgroups.

## 2. Results

### 2.1. PODNL1 Expression and Methylation in LGG

**Figure 1:**
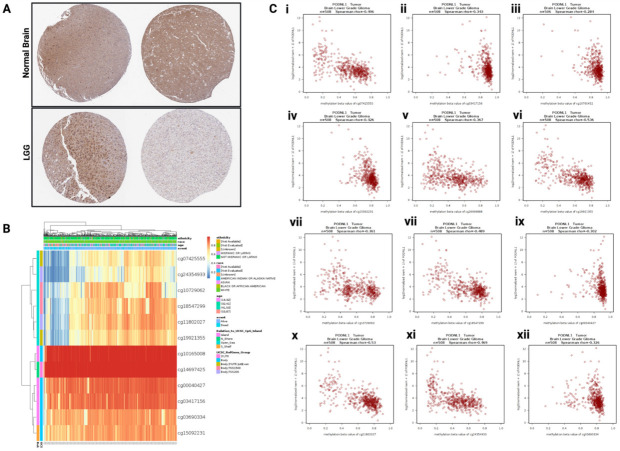
*PODNL1* protein expression, CpG methylations, and its correlation with *PODNL1* mRNA levels. (**A**) Immunohistochemical images showing *PODNL1* protein expression in normal brain and lower-grade glioma (LGG) tissues (Human Protein Atlas). Weak *PODNL1* expression in glial cells and moderate expression in neuronal cells were reported in normal brain tissues. Moderate expression was reported for one LGG sample (left), while weak expression was reported in the other (right). (**B**) Heatmap showing methylation levels in the top twelve *PODNL1* CpG loci in TCGA-LGG (MethSurv). (**C**) (**i**–**xii**.) Correlation between TCGA-LGG *PODNL1* CpG methylations (beta-values) and *PODNL1* mRNA expressions (RSEM+1) (*p* < 0.05 for all). Spearman’s rho correlation coefficients are reported within each figure.

To investigate the differential expression of *PODNL1* in LGG, *PODNL1* protein expression in normal brain tissues and LGG tissues was compared using immunohistochemical data from the Human Protein Atlas (Figure 1A). Normal brain tissues showed moderate expression of *PODNL1* protein in the neuronal cells and weak expression in glial cells. In the two LGG samples analyzed, *PODNL1* protein was underexpressed in LGG compared with the two normal brain tissues. Notably, one LGG sample showed particular underexpression of *PODNL1* protein compared with the other LGG sample. Thus, intertumoral heterogeneity of *PODNL1* expression may exist between LGG samples, depending on unknown factors, and it is possible that there are subgroups of LGGs with high and low *PODNL1* expression. As *IDH1* mutation, which is highly frequent in up to 70–90% LGGs [11,12], plays a role in inducing global DNA hypermethylation [6], we investigated the methylation levels of *PODNL1* CpG islands in TCGA-LGG and found hypermethylation in multiple CpGs in this gene. Methylation patterns of the twelve most hypermethylated *PODNL1* CpGs are visualized in Figure 1B. To further examine the potential role of *PODNL1* CpG methylations in regulating *PODNL1* expression, we determined the correlation between CpG site methylation levels (beta-values) and *PODNL1* mRNA expression (RSEM+1). Significant negative correlations between *PODNL1* CpG methylations and mRNA expressions were observed in multiple cases with Spearman’s rho correlation coefficients ranging from −0.072 to −0.53 (*p* < 0.05). Scatterplots of CpG methylations correlating with *PODNL1* mRNA with a Spearman’s rho correlation coefficient of −0.28 or below are presented in Figure 1(Ci–Cxii), while data for other CpGs with weaker correlation coefficients are presented in Supplementary Figure S1. These results indicate that methylation of *PODNL1* CpG sites is potentially involved in the downregulation of *PODNL1* mRNA expressions in varying degrees in LGG.

### 2.2. Association between PODNL1 Aberrations and Tumor Aggressiveness in LGG

**Figure 2:**
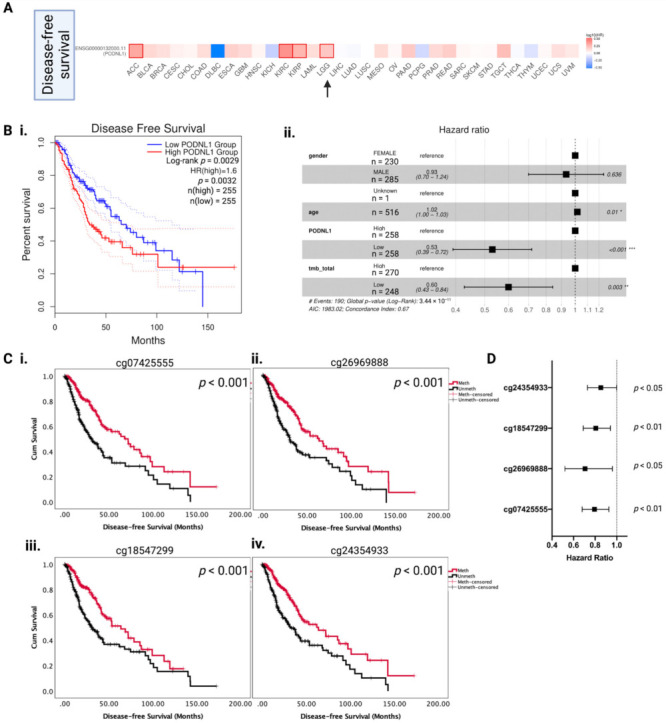
*PODNL1* mRNA expression and methylation associates with LGG disease-free survival (DFS). (**A**) Log_10_ hazard ratio heatmap showing the association between *PODNL1* mRNA expression and DFS across TCGA cancers. Solid outlined border represents statistically significant prognostic effects (*p* < 0.05). Lower-grade glioma (LGG) is indicated with a black arrow. (**B**) (**i**). Kaplan–Meier survival curve showing DFS based on *PODNL1* mRNA levels (median expression cut-off). The curves show 95% confidence intervals for each group in dotted lines. (**ii**). Forest plot showing multivariate hazard ratio based on gender, age, tumor mutational burden (TMB), and *PODNL1* mRNA expression groups (median expression cut-off) for TCGA-LGG. *** *p* < 0.001, ** *p* < 0.01 and * *p* < 0.05. (**C**) (**i**–**iv**). Kaplan–Meier survival curves (DFS) based on methylation levels (median beta value cut-off) of four *PODNL1* CpGs. The red line indicates “Meth” or the high methylation group and the black line indicates “Unmeth” or the low methylation group. Sample numbers *n* = 254 for each methylation group, for all figures. Log-rank *p*-values are marked in each figure. A *p*-value of <0.05 was considered significant. (**D**) Forest plot of DFS multivariate analysis hazard ratios for each significant CpG, after adjusting for age, gender, tumor grade, and *IDH1* mutation.

Previously, *PODNL1* mRNA overexpression was reported to be associated with unfavorable OS in LGG and glioblastoma [19]. As DFS indicates tumor aggressiveness through faster recurrence, we firstly analyzed the prognostic effects of *PODNL1* mRNA in the DFS of all TCGA cancers (Figure 2A), where *PODNL1* mRNA overexpression was associated with significantly unfavorable DFS in four TCGA cancers including ACC, KIRC, KIRP, and LGG. These prognostic effects in LGG DFS were further examined with Kaplan–Meier survival analysis where the high *PODNL1* mRNA group showed a median DFS of 30 months versus 60 months in the low *PODNL1* mRNA group (*p* < 0.01; Figure 2Bi). Multivariate survival analysis confirmed the independent unfavorable prognostic effects of *PODNL1* mRNA overexpression in LGG (Figure 2Bii). As we found a significant association between methylation of *PODNL1* CpG sites and mRNA expression, we investigated whether the methylation of these particular CpGs affects the *PODNL1* prognostic effects, and performed univariate and multivariate survival analysis (DFS and OS) adjusting for patient age, gender, tumor grade, and *IDH1* mutation status (Table 1).

We found that 15 CpG methylations were associated with significantly longer OS, of which 12 CpG methylations were associated with significantly longer DFS in univariate analysis. In multivariate analysis, 7 CpG (cg07425555, cg19921355, cg24354933, cg11802027, cg11453058, cg26969888, and cg18547299) methylations significantly affected LGG OS, of which 4 CpG methylations (cg07425555, cg26969888, cg18547299, and cg24354933) were significant prognostic factors in LGG DFS. The Kaplan–Meier survival curves for CpGs that are significantly prognostic after adjusting for age, gender, tumor grade, and *IDH1* mutation are presented in Figure 2(Ci–Civ) for DFS along with forest plot visualization of hazard ratios with 95% CI in Figure 2D. For cg07425555 (Figure 2Ci), the median DFS of high methylation (“Meth”) versus low methylation (“Unmeth”) was 72 (52–91) months versus 31 (21–40) months (*p* < 0.001). For cg26969888, the median DFS of Meth versus Unmeth was 63 (44–83) months versus 30 (22–37) months (*p* < 0.001). For cg18547299, the median DFS of Meth versus Unmeth was 63 (44–82) months versus 31 (22–39) months (*p* < 0.001). For cg24354933, the median DFS of Meth versus Unmeth was 63 (41–86) months versus 32 (25–40) months (*p* < 0.001). Thus, between these CpGs, the median DFS ranged from 63 to 72 months for Meth versus 31 to 32 months for Unmeth, and these significant differences in DFS confirm the association between low methylation in these CpG sites and increased tumor aggressiveness.

We further investigated the association between these *PODNL1* CpG methylation levels (beta-values) and the histological subtypes and grades of LGG (Supplementary Figure S2). In oligodendroglioma, there was no significant difference in CpG methylation levels between grade II and grade III for all four CpGs. In astrocytoma, CpG methylation levels were significantly lower in grade III compared with grade II for all four CpGs (*p* < 0.001). This indicates the possible demethylation of *PODNL1* during grade progression and its role in astrocytoma aggressiveness.

**Figure 3:**
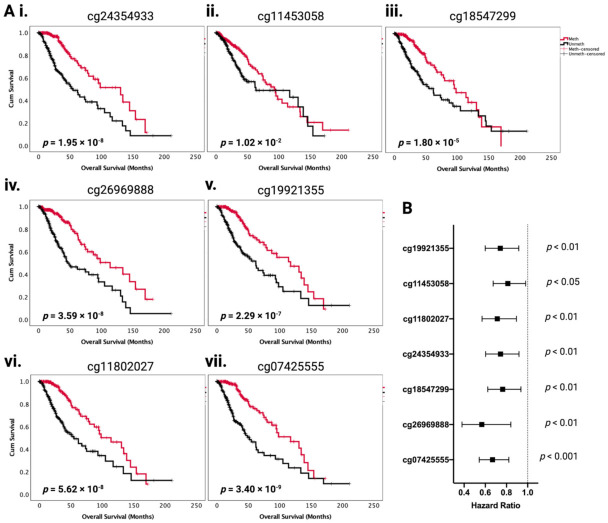
*PODNL1* CpG methylation status affects LGG overall survival (OS). (**A**) (**i**–**vii**). Kaplan–Meier survival curves for LGG OS based on the methylation status of specific *PODNL1* CpGs (median beta value cut-off). The red line indicates “Meth” or high methylation group and the black line indicates “Unmeth” or low methylation group. Log-rank *p*-values are marked in each figure. A *p*-value of <0.05 was considered significant. Sample numbers *n* = 254 for each methylation group, for all figures. (**B**) Forest plot of OS multivariate analysis hazard ratios for each significant CpG, after adjusting for age, gender, tumor grade, and IDH1 mutation.

These prognostic effects in DFS are mirrored in the OS analysis, where cg07425555 Meth showed a median OS of 117 (84–150) months versus 52 (35–68) months in Unmeth, cg26969888 Meth showed 114 (71–156) months versus 46 (27–64) in Unmeth, cg18547299 Meth showed 98 (63–132) months versus 61 (39–84) in Unmeth, and cg24354933 Meth showed 130 (70–190) months versus 54 (37–72) months in Unmeth (*p* < 0.001 or *p* < 0.01 for all). Three additional CpG sites compared with DFS were significant prognostic factors for OS in multivariate analysis, which also showed the same prognostic patterns (Figure 3((Ai–Avii); Table 1). The hazard ratios with 95% CI are visualized in a forest plot in Figure 3B. Within the *IDH1* mutant LGG histological subtypes analysed separately for grade II and grade III tumours (Supplementary Figure S3), cg26969888 was a significant prognostic factor for OS in both grade II and grade III oligodendroglioma, as well as in grade III astrocytoma. In grade II *IDH1* mutant astrocytoma, cg07425555 and cg24354933 were significant prognostic factors for OS. Our results are concordant with and validate the known association between *PODNL1* mRNA overexpression and unfavorable OS in glioma [19], as we show that methylation of *PODNL1* is associated with decreased *PODNL1* expression, and specific *PODNL1* CpG methylations are associated with significantly improved OS. Thus, *PODNL1* methylation is an important occurrence in LGG, which affects disease aggressiveness and clinical outcome. In particular, a set of four CpGs (cg07425555, cg26969888, cg18547299, and cg24354933) may be of importance, as they show significant independent prognostic effects in both OS and DFS, after adjustments for age, gender, tumor grade, and *IDH1* mutation in multivariate analysis.

### 2.3. A Subset of LGGs with Low PODNL1 Methylation Is Associated with Increased Immune Cell Infiltration in the Tumor Microenvironment

**Figure 4:**
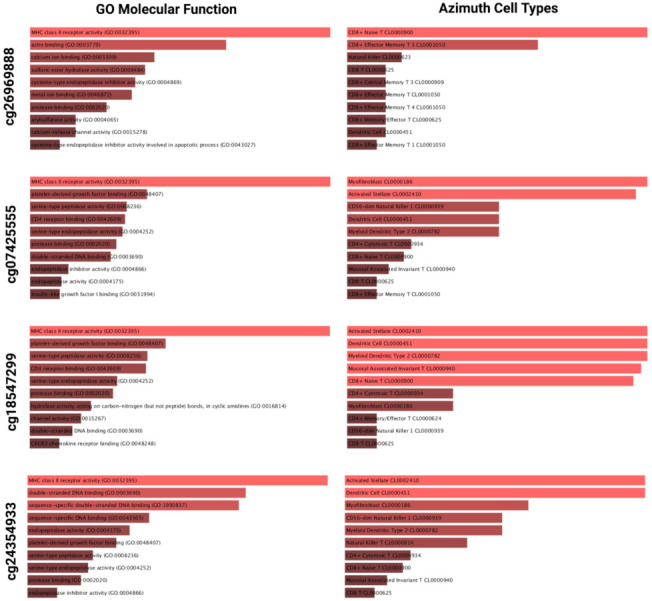
Gene Ontology (GO) molecular function and Azimuth cell type analysis using the top 1000 significantly upregulated genes (Benjamini–Hochberg false discovery rate < 0.01) in PODNL1 low methylation groups of four significant CpGs. Data represents −log10 *p*-values. All enriched terms have *p*-values < 0.05.

To elucidate the possible mechanisms associated with the increased tumor aggressiveness in the low methylation of specific CpGs, we determined the differentially expressed genes (DEGs) between high and low methylated groups of each of the four significant CpGs that showed significant prognostic effects in the OS and DFS of LGGs in multivariate analysis. The top 1000 genes upregulated in the low methylation group of each CpG were analyzed for enriched GO term, molecular pathways, and cell types. The enriched GO molecular functions and Azimuth cell types are presented in Figure 4 (the corresponding data for the high methylation group are presented in Supplementary Figure S4). For all four CpGs, the low methylation group was most significantly associated with the GO term enrichment of MHC class II receptor activity (GO:0032395), and for two CpGs, there was enrichment of CD4 receptor binding (GO:0042609) (Figure 4). The cell types enriched in these low methylation groups were predominantly immune cells for all CpGs, including different types of T cells, dendritic cells, and natural killer cells (Figure 4). In contrast, in the groups with high methylation of these CpGs, significant enrichments related to RNA binding (GO:0003723) and ligand-gated channel activity (GO:0022834), and cell types related to oligodendroglial precursor cell (CL0002453) were present (Supplementary Figure S4).

WikiPathway and KEGG pathway analysis of the DEGs showed significant enrichments of inflammatory response pathway (WP453) and other immune-related pathways in all low methylation groups of each CpG type (Supplementary Figure S5). In the high methylation groups, significant enrichments of pathways involved nicotine addiction, neuro-active ligand–receptor interaction, ribosome, and other non-oncogenic pathways. Descartes cell type analysis showed enrichment of lymphoid cells and stromal cells in low methylation groups for all CpGs, and neurons and oligodendrocytes in the high methylation groups for all CpGs (Supplementary Figure S5).

Collectively, these results indicate that, in LGGs with low methylation levels of these CpGs, there may be enrichments of immune cells in the tumor microenvironment and activated inflammatory response pathways. Whereas, in LGGs with high methylation levels of these CpGs, non-oncogenic cells and functions are enriched. These results support the findings of unfavorable survival associated with low methylation of these CpGs in LGG, as immunity-high LGGs have been reported to show higher tumor stemness and epithelial–mesenchymal transition scores, leading to unfavorable survival [21,22].

**Figure 5:**
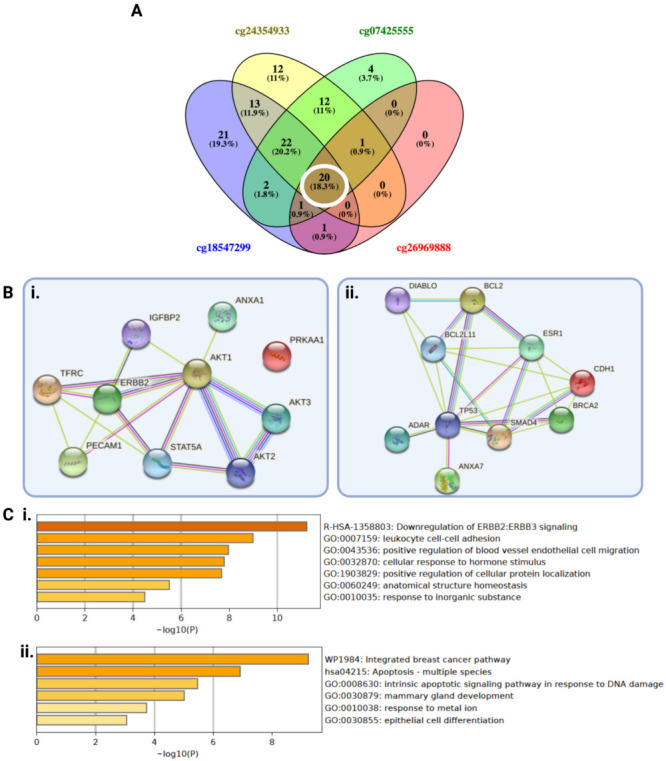
Differential protein expression, protein–protein interaction (PPI), and enrichment analysis. (**A**) Venn diagram presenting the overlap between differentially expressed proteins in four significant *PODNL1* CpGs. The white circle represents common differentially expressed proteins between all four CpGs, used for further analysis. (**B**) PPI network visualization of ten proteins upregulated in (**i**). low CpG methylation groups and (**ii**). high CpG methylation groups (STRING). (**C**) Enrichment analysis (MetaScape) of ten upregulated proteins in (**i**). low CpG methylation groups and (**ii**). high CpG methylation groups. Data represents −log_10_ *p*-values. All enriched terms have *p*-values < 0.05.

As significantly differentially expressed proteins and PPI network analysis can shed light on the protein networks involved in the different behavior of the tumor, we identified twenty common differentially expressed proteins that are significantly upregulated in the low methylation groups (ten proteins) and the high methylation groups (ten proteins) of all four CpGs (Figure 5A). The identification of these common proteins upregulated in the low methylation group suggests that the mechanism of prognostication may be similar among all four significant CpGs. We performed and visualized the PPI network analysis for upregulated proteins in low and high groups separately in STRING (Figure 5(Bi,Bii)), which showed the significant interactions between these proteins.

Enrichment analysis of upregulated proteins in low methylation groups of CpGs showed the most significant GO term enrichment of leukocyte cell–cell adhesion (Figure 5Ci), which is associated with immune response, thus reflecting the findings from DEGs’ analysis. Apoptosis, apoptosis signalling pathway in response to DNA damage, and epithelial cell differentiation were enriched in the high methylation group, potentially reflecting the inherent lower aggressiveness of these LGGs (Figure 5Cii). These results further support the survival analysis and DEG analysis, and suggest the association between immune cell infiltration and low *PODNL1* methylation groups.

The PPI network of AKT1, AKT2, AKT3, ERBB2, and STAT5A was particularly strongly connected in the low *PODNL1* methylation group (Figure 5Bi) and there was enrichment of the reactome pathway, upstream of the AKT signaling pathway, related to downregulation of ERBB2/ERBB3 signaling (Figure 5Ci).

**Figure 6:**
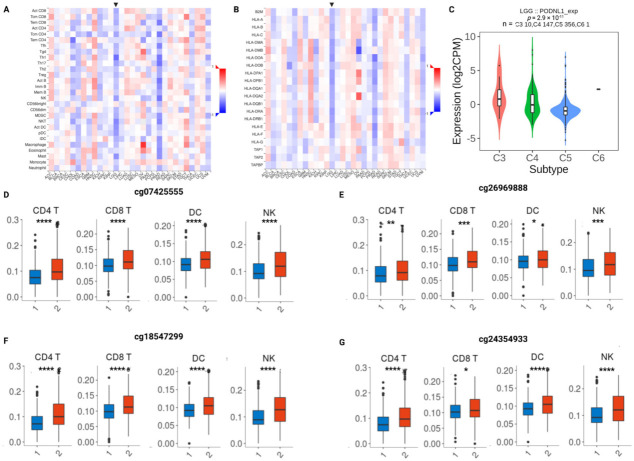
Association of *PODNL1* methylation and expression, with immune cell infiltration and immune subtypes. Correlation heatmap between *PODNL1* methylation (beta values) and (**A**) immune infiltrates and (**B**) MHC class II molecules across TCGA cancers (LGG marked with a black arrow; analyzed using TISIDB). (**C**) *PODNL1* mRNA expression levels in TCGA-LGG immune subtypes. C3: inflammatory (*n* = 10), C4: lymphocyte depleted (*n* = 147), C5: immunologically quiet (*n* = 356), C6: TGF-b dominant (*n* = 1). One-way ANOVA *p*-value = 2.9 × 10^−11^. (**D**–**G**) Association between specific *PODNL1* CpG methylation group (group 1 = high methylation and group 2 = low methylation; stratified by median beta value cut-off) infiltrating immune cells in all four significant CpGs. Statistical significance was determined by a Student’s t-test with a *p*-value < 0.05. **** *p* < 0.0001, *** *p* < 0.001, ** *p* < 0.01, and * *p* < 0.05.

We explored the association between *PODNL1* methylation levels and a range of immune cell infiltration levels across TCGA cancers (Figure 6A). A clear negative correlation between immune cell infiltration and *PODNL1* methylation in LGG and PRAD was observed. A significant negative correlation between LGG *PODNL1* methylation and 27 out of 28 immune cells was observed (Figure 6A). We found enrichment of immune-related MHC class II receptor activity in the DEG analysis, thus we also investigated the correlation between *PODNL1* methylation and MHC class II molecules (Figure 6B). In accordance with the other findings, we observed a significant negative correlation between *PODNL1* methylation and all 21 MHC class II molecules analyzed (Figure 6B). These findings suggest that *PODNL1* may play a role in immune infiltration in LGG; however, it may not be involved in the immune microenvironment of other cancers. Further studies incorporating tumor histological and molecular subtypes and tumor grades are required to investigate its potential immunomodulatory role in other cancers.

We found a significant association between LGG immune subtypes [23] and *PODNL1* mRNA expression, where high *PODNL1* mRNA expression (potentially corresponding to low methylation) was associated with “C3: inflammatory” subtype, moderate *PODNL1* mRNA expression was associated with “C4: lymphocyte depleted” subtype, and low *PODNL1* mRNA expression was associated with “C5: immunologically quiet” subtype (Figure 6C).

A significant association between immune cell infiltration and all of the four prognostic CpGs were observed for multiple immune infiltrates including CD4+ T cells, CD8+ T cells, dendritic cells, and natural killer cells (Figure 6D–G). Data for other immune infiltrates are presented in Supplementary Figure S6. In all significant observations, the immune infiltrate level was higher in lower methylation groups of these CpGs.

We further investigated the immune cell infiltration levels of 24 immune cell types within *PODNL1* high/low oligodendroglioma and astrocytoma separately for all four CpGs, and found that there was increased immune cell infiltration in the low methylation groups of these CpGs within both LGG subtypes (Supplementary Figure S7 and S8). In astrocytoma, the majority of the 24 immune cell types were significantly increased in the low methylation subgroups of the four CpGs, whereas in oligodendroglioma, the number of infiltrating immune cell types was lower than in astrocytoma, and depended on the specific CpG site.

Collectively, these results strongly support the potential high immune cell infiltration and inflammatory response activity in LGGs with low *PODNL1* methylation levels. It is possible that the observed unfavorable prognosis and tumor aggressiveness associated with low *PODNL1* methylation/high *PODNL1* expression is related to the enrichment of immune cell infiltration in the tumor microenvironment, as previous reports identified shorter glioma survival in patients with high immune infiltration [21,22,23,24,25].

### 2.4. PODNL1 Methylation May Affect Immune Checkpoint Blockade Response in LGG

**Figure 7:**
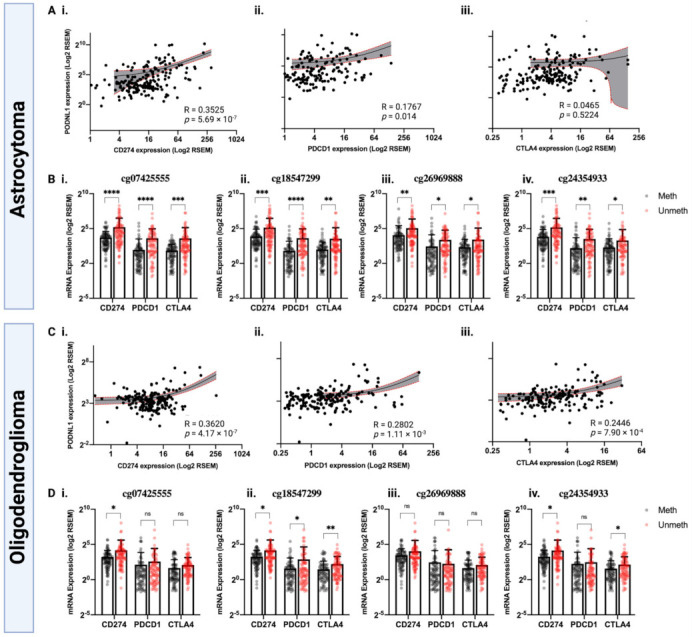
Association between *PODNL1* expression/specific *PODNL1* CpG methylations and immunotherapeutic targets—*PD-L1* (*CD274*), *PD-1* (*PDCD1*) and *CTLA4*, in TCGA astrocytoma (**Ai**–**Aiii**) and (**Bi**–**Biv**) and in TCGA oligodendroglioma (**Ci**–**Ciii**) and (**Di**–**Div**). R = Spearman’s rho correlation coefficient. Methylation groups of high (meth) and low (unmeth) were determined with a median beta-value cutoffs for each CpG. A *p*-value of less than 0.05 was considered statistically significant. **** *p* < 0.0001, *** *p* < 0.001, ** *p* < 0.01, * *p* < 0.05, and ns = not significant.

As the methylation status of specific *PODNL1* CpGs showed strong associations with immune cell infiltration, we investigated the potential efficacy of immune checkpoint blockade (*PD-L1, PD-1,* and *CTLA4*) in groups of TCGA-LGG histological subtypes, astrocytoma and oligodendroglioma, based on *PODNL1* expression and CpG methylation status (Figure 7). In astrocytoma, *PODNL1* expression showed significant positive correlations with PD-1 and PD-L1 expression (Figure 7(Ai,Aii)). Low methylated groups of all of the four *PODNL1* CpG sites showed significantly increased expression of *PD-1, PD-L1*, and *CTLA4*, compared with the high methylated groups (Figure 7(Bi–Biv)). In oligodendroglioma, there was significant positive correlation between *PODNL1* expression and the expression of all three immunotherapeutic targets (Figure 7(Ci–Ciii)). The association between *PODNL1* CpG methylation status and the three immunotherapeutic targets in oligodendroglioma depended on the specific CpG sites, with no association observed for cg26969888, significantly higher expression of only *PD-L1* in low methylated cg07425555, significantly higher expression of *PD-L1* and *CTLA4* in low methylated cg24354933, and significantly higher expression of all three targets in low methylated cg18547299 (Figure 7(Di–Div)).

Collectively, these results indicate that *PODNL1* methylation may affect immune checkpoint blockade therapy response in both astrocytoma and oligodendroglioma in varying degrees. Additionally, the methylation levels of cg07425555, cg26969888, cg18547299, and cg24354933 can potentially serve as biomarkers for strategizing immune checkpoint blockade therapy in LGG.

## 3. Discussion

In this study, we investigated the potential role of an SLRP class V member, *PODNL1*, in determining LGG aggressiveness. It is a largely uncharacterized and understudied gene; however, its high expression in high-grade glioma [18] and its role as a prognostic factor in high and low-grade gliomas [19,26] and ovarian cancers [27] warranted a comprehensive analysis of its influence in LGG aggressiveness, which this study has conducted. We have found that *PODNL1* overexpression is associated with a significantly more unfavorable DFS, thus *PODNL1* overexpressed LGGs recurred at a faster rate than underexpressed *PODNL1*. This finding complements the OS analysis carried out by Geng et. al., where *PODNL1* overexpression was associated with a significantly shorter OS [19].

As little is known about *PODNL1* mRNA regulation, we investigated the role of *PODNL1* methylation in regulating gene expression, particularly owing to the high frequency of *IDH1* mutation in LGGs [11], which is associated with global DNA hypermethylation [6]. We reported multiple CpGs that showed significant negative correlations with *PODNL1* mRNA expression in the TCGA-LGG dataset. CpG methylation has been commonly associated with gene expression silencing in normal and cancer cells [28,29,30,31]. Thus, it is possible that *PODNL1* mRNA expression is epigenetically regulated in LGG. A comprehensive survival analysis revealed four *PODNL1* CpGs (cg07425555, cg26969888, cg18547299, and cg24354933) that were significant independent prognostic factors for both OS and DFS in multivariate analysis (adjusted for age, gender, tumor grade, and *IDH1* mutation status), where low methylation levels were associated with a more unfavorable clinical outcome. Methylations in these CpGs negatively correlate with *PODNL1* mRNA expression in LGG, thus our findings are concordant with the previous report of improved OS in *PODNL1*-underexpressed LGGs [19]. Furthermore, their significant associations with LGG DFS suggest that low methylation of these CpGs may contribute to LGG aggressiveness. The relationship between global demethylation patterns in recurrent gliomas and tumor aggressiveness has been reported previously [32], and particular demethylation patterns were noted in tumors that exhibited malignant transformation [33]. However, the influences of individual genes methylations, and particularly specific CpG methylations, are important to be elucidated in order to potentially find therapeutic targets, as the prognostic roles of particular gene methylations may be opposing in nature, complicating the efficacies of demethylating therapeutic agents such as 5-Azacytidine [34,35,36,37,38,39]. Our study elucidates the importance of specific *PODNL1* CpG methylations in LGG aggressiveness, which may potentially facilitate the development of targeted therapy.

Our findings from the differential gene and protein expression analysis suggest that the aggressive tumor behavior in hypomethylated *PODNL1* CpGs may involve increased inflammatory pathways and immune cell infiltration levels. Further analysis of the association between the four significant CpGs and immune cell infiltration levels confirmed the significant enrichment of a range of immune infiltrates in low-methylated groups of all four CpGs. Specific CpG methylations have previously been associated with immune cell infiltration levels (thoroughly reviewed by Bacolod et. al, 2020) [40]. Increased immune cell infiltration levels are linked to heightened tumor aggressiveness in a range of cancers including breast cancer, kidney renal clear cell carcinoma, uveal melanoma, pancreatic cancer, and osteosarcoma [41,42,43,44,45,46,47,48]. In gliomas, including LGG, increased immune cell infiltration levels showed poor prognosis and more aggressive phenotypes [21,22,49,50,51,52,53,54,55,56,57]. Thus, we report that low *PODNL1* methylation, specifically of CpG sites cg07425555, cg26969888, cg18547299, and cg24354933, may be a key factor in the modulation of immune infiltrates in the LGG tumor microenvironment, affecting its aggressiveness and prognosis.

Another factor that may determine LGG aggressiveness in low methylation groups of these *PODNL1* CpGs could involve the AKT signaling pathway, which we found to be enriched in this group, as previous studies reported the association between phosphorylation of AKT and glioma aggressiveness [58,59,60,61].

Lastly, we showed that high *PODNL1* expression subgroups and low methylation subgroups of the identified *PODNL1* CpGs within the histological subtypes of LGG may be particularly responsive to immune checkpoint blockade therapy; however, in vitro and in vivo studies are warranted to confirm these findings. Thus, *PODNL1* CpG methylation may also be a potential biomarker for LGG immune subtypes and immune checkpoint blockade response prediction.

## 4. Materials and Methods

### 4.1. Tumor Cohort

The Cancer Genome Atlas (TCGA) lower-grade glioma (LGG) was used for analysis in this study. Clinical and mutational data for the LGG tumors were downloaded from the cBioPortal platform (www.cbioportal.org, accessed on 7 September 2021). Duplicate samples were removed where more than one sample existed for a patient, and the total cohort size with the required clinical information available was *n* = 508. The median age of the TCGA-LGG cohort was 41 (14–87) and the male/female ratio was 1.2/1.

### 4.2. PODNL1 Expression and Methylation Analysis

Normal brain and LGG *PODNL1* protein expression in the form of immunohistochemistry data was queried and retrieved from the Human Protein Atlas (www.proteinatlas.org, accessed on 15 September 2021).

*PODNL1* methylation data were explored in MethSurv [62] and TCGA-Wanderer [63] to identify commonly methylated *PODNL1* CpG sites in LGG. CpG site methylations with statistically significant correlation (*p* < 0.05) with *PODNL1* mRNA expression were identified through correlation scatterplots in TCGA-Wander [63]. Methylation beta values for each *PODNL1* CpG site in TCGA LGG samples, which significantly correlated with *PODNL1* mRNA, were downloaded from SMART App [64].

### 4.3. Survival Analysis

Disease-free survival (DFS) was termed in this study as the period of time from the patient’s primary treatment end until the date of relapse or censored at last follow-up. Overall survival (OS) was termed as the period of time the patient survived from the date of diagnosis until the date of death (or censored at the last follow update). Survival analysis was performed using the Kaplan–Meier survival method and Cox proportional hazard’s model. Statistical significance was determined by a log-rank *p*-value of less than 0.05.

Survival analysis of DFS based on *PODNL1* mRNA expression (median cut-off) was performed in GEPIA2.0 [65]. Multivariate analysis to determine independent prognostic effects of *PODNL1* mRNA expression was performed in CVCDAP [66]. The prognostic effects of *PODNL1* mRNA expression across all TCGA cancers were visualized in a heatmap using GEPIA2.0 [65].

For survival analysis (DFS and OS) based on individual methylated CpG sites, median beta-value was used as a cut-off for each methylated CpG site. IBM SPSS version 27 was used to perform Kaplan–Meier and Cox proportional analysis. Multivariate analysis adjusting for age, gender, tumor grade, and *IDH1* mutation status was performed in IBM SPSS using Cox proportional hazard’s model. Hazard ratios with 95% confidence interval (95% CI) that are statistically significant were visualized in forest plots generated using GraphPad Prism version 9.

### 4.4. Differential Gene and Protein Expression Analysis

Median beta value cut-off was used for each prognostically significant CpG site to stratify the TCGA samples into two groups with high (methylated) and low (unmethylated) *PODNL1* methylation. Custom groups were created in the cBioPortal platform by importing TCGA sample IDs of methylated and unmethylated groups for each CpG site, separately. Methylated and unmethylated groups for each CpG site were analyzed and compared in cBioPortal to identify significantly differentially expressed genes. The top 1000 most upregulated genes in methylated and the top 1000 most upregulated genes in unmethylated groups were identified with *p*-values corrected for multiple comparisons using the Benjamini–Hochberg procedure false discovery rate (FDR < 0.01). The gene lists were imported to EnrichR platform [67] for Gene Ontology (GO) term, pathway, and cell-type enrichment analysis in methylated and unmethylated groups separately. GO cellular component, molecular function and biological process, KEGG pathway, WikiPathways, Descartes cell types, and Azimuth cell types enrichments were analyzed for each group of CpGs.

Differential protein expression (*p*-values corrected for multiple comparisons using the Benjamini–Hochberg procedure; FDR < 0.05) between groups of methylated and unmethylated CpGs (for four significant CpGs) was downloaded from cBioPortal, and proteins that were differentially expressed between methylated and unmethylated groups for all four CpGs were curated. The protein–protein interaction (PPI) network was generated and visualized using STRING (www.string-db.org, accessed on 16 September 2021) by importing the curated list of differentially expressed genes. Proteins upregulated in unmethylated groups and methylated groups were analyzed separately in MetaScape [68] to determine significant GO terms and pathway enrichments within each group.

### 4.5. Immune Cell Infiltration Analysis

Correlations between *PODNL1* methylation and immune cell infiltration levels and MHC class II molecules were analyzed using TISIDB [69]. Associations between individual CpG methylation and immune cell infiltration levels were analyzed using LGG transcriptomics data retrieved from cBioPortal and ImmuCellAI [70]. *PODNL1* mRNA expression within each immune subtype [23] of TCGA LGG was visualized and analyzed in TISIDB [69].

### 4.6. Immune Checkpoint Blockade (ICB) Therapy Response Prediction

TCGA-LGG transcriptomics data were retrieved from cBioPortal, and expression data for *PODNL1, PD-1, PD-L1,* and *CTLA4* were extracted for astrocytoma and oligodendroglioma samples. Methylation beta values for each CpG site were retrieved from SMART App [64]. Using beta value median cut-off, the TCGA-LGG histological subtype samples were stratified by “meth” (high methylation) and “unmeth” (low methylation) groups of CpG sites. Correlation and association between PODNL1 expression and PODNL1 CpG methylation, respectively, were then analyzed in GraphPad Prism v9 (San Diego, CA, USA).

## Figures and Tables

**Table 1:** Univariate and multivariate survival analysis based on *PODNL1* CpG methylation high/low subgroups.

		Univariate Analysis				Multivariate Analysis			
		Overall Survival		Disease-Free Survival		Overall Survival		Disease-Free Survival	
CpG loci	Position	HR [95% CI]	*p*-Value	HR [95% CI]	*p*-Value	HR [95% CI]	*p*-Value	HR [95% CI]	*p*-Value
*cg26498537*	13929563	0.68 [0.48–0.97]	3.40 × 10^−2^	0.75 [0.56–1.00]	5.26 × 10^−2^	1.01 [0.69–1.47]	9.60 ×10^−1^	0.95 [0.70–1.28]	7.68 × 10^−1^
*cg21993464*	13930493	0.60 [0.42–0.87]	6.56 × 10^−3^	0.82 [0.62–1.09]	1.89 × 10^−1^	0.91 [0.63–1.33]	6.57 × 10^−1^	0.99 [0.74–1.33]	9.82 × 10^−1^
*cg14697425*	13932027	1.02 [0.72–1.46]	8.81 × 10^−1^	1.05 [0.79–1.39]	7.23 × 10^−1^	1.20 [0.83–1.73]	3.12 × 10^−1^	1.19 [0.89–1.60]	2.25 × 10^−1^
*cg03417156*	13932833	0.66 [0.46–0.94]	2.34 × 10^−2^	0.78 [0.58–1.04]	9.42 × 10^−2^	1.09 [0.73–1.62]	6.50 × 10^−1^	1.07 [0.79–1.45]	6.47 × 10^−1^
*cg03690334*	13933004	0.57 [0.40–0.82]	2.43 × 10^−3^	0.72 [0.54–0.96]	2.62 × 10^−2^	0.72 [0.49–1.04]	8.64 × 10^−2^	0.85 [0.63–1.14]	2.93 × 10^−1^
*cg10165008*	13933098	0.60 [0.42–0.86]	6.25 × 10^−3^	0.72 [0.54–0.96]	2.83 × 10^−2^	0.83 [0.57–1.21]	3.45 × 10^−1^	0.83 [0.61–1.12]	2.40 × 10^−1^
*cg00040427*	13933384	0.43 [0.29–0.62]	6.00 × 10^−6^	0.59 [0.44–0.79]	4.20 × 10^−4^	0.71 [0.48–1.06]	1.01 × 10^−1^	0.79 [0.58–1.08]	1.54 × 10^−1^
*cg15092231*	13935853	0.49 [0.34–0.71]	1.49 × 10^−4^	0.65 [0.49–0.87]	3.66 × 10^−3^	0.79 [0.53–1.18]	2.62 × 10^−1^	0.87 [0.64–1.18]	3.82 × 10^−1^
*cg07425555*	13938206	0.32 [0.22–0.46]	3.40 × 10^−9^	0.47 [0.35–0.63]	5.45 × 10^−7^	0.44 [0.29–0.67]	1.39 × 10^−4^	0.62 [0.46–0.85]	3.53 × 10^−3^
*cg19921355*	13938280	0.37 [0.25–0.54]	2.29 × 10^−7^	0.56 [0.42–0.74]	8.30 × 10^−5^	0.54 [0.35–0.84]	5.70 × 10^−3^	0.76 [0.55–1.05]	1.03 × 10^−1^
*cg24354933*	13938482	0.33 [0.22–0.49]	1.95 × 10^−8^	0.51 [0.38–0.68]	7.00 × 10^−6^	0.55 [0.36–0.84]	5.93 × 10^−3^	0.72 [0.53–0.99]	4.74 × 10^−2^
*cg11802027*	13938630	0.35 [0.24–0.51]	5.62 × 10^−8^	0.53 [0.39–0.70]	1.70 × 10^−5^	0.50 [0.32–0.79]	3.32 × 10^−3^	0.76 [0.54–1.07]	1.25 × 10^−1^
*cg18547299*	13938766	0.44 [0.30–0.64]	1.80 × 10^−5^	0.52 [0.39–0.70]	1.50 × 10^−5^	0.58 [0.38–0.87]	9.25 × 10^−3^	0.64 [0.47–0.88]	6.52 × 10^−3^
*cg10729062*	13939010	0.48 [0.33–0.69]	1.05 × 10^−4^	0.50 [0.37–0.67]	5.00 × 10^−6^	0.73 [0.48–1.10]	1.37 × 10^−1^	0.73 [0.53–1.01]	5.73 × 10^−2^
*cg11453058*	13953364	0.62 [0.44–0.89]	1.02 × 10^−2^	0.70 [0.53–0.94]	1.73 × 10^−2^	0.66 [0.45–0.96]	3.03 × 10^−2^	0.79 [0.59–1.06]	1.18 × 10^−1^
*cg26969888*	13953442	0.34 [0.24–0.50]	3.59 × 10^−8^	0.51 [0.38–0.68]	7.00 × 10^−6^	0.56 [0.38–0.84]	4.85 × 10^−3^	0.70 [0.51–0.96]	2.65 × 10^−2^
*cg10760452*	13953776	0.72 [0.50–1.02]	7.00 × 10^−2^	0.83 [0.62–1.10]	2.07 × 10^−1^	0.79 [0.55–1.13]	2.06 ×10^−1^	0.94 [0.71–1.27]	7.26 × 10^−1^
*cg10609371*	13954060	0.94 [0.66–1.34]	7.38 × 10^−1^	1.04 [0.78–1.39]	7.50 × 10^−1^	1.14 [0.79–1.65]	4.60 × 10^−1^	1.14 [0.85–1.52]	3.68 × 10^−1^

## Data Availability

All data accessed in this study are available on publicly available databases as described in Section 4.

## Supplementary Materials

The following are available online at https://www.mdpi.com/article/10.3390/ijms222212572/s1.
