# Supplementary material for: PODNL1 Methylation Serves as a Prognostic Biomarker and Associates with Immune Cell Infiltration and Immune Checkpoint Blockade Response in Lower-Grade Glioma
Source: Int J Mol Sci. 2021 Nov 22;22(22):12572. doi: 10.3390/ijms222212572 (PMC8625785; doi:10.3390/ijms222212572)
Supplement: Supplementary file 1 [file ijms-22-12572-s001.zip › ijms-1444137-supplementary.pdf]

Supplementary Figures

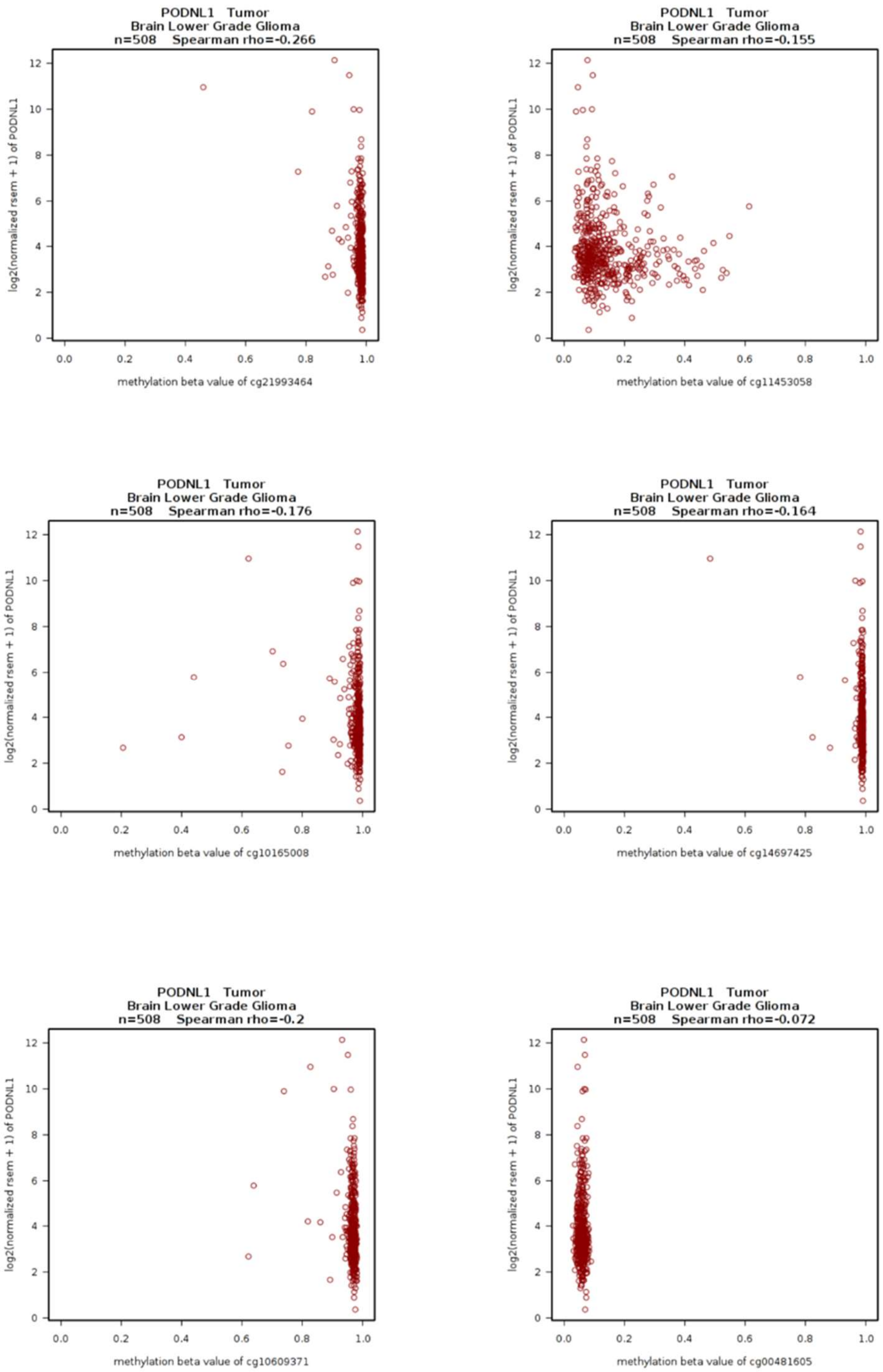

Figure S1. Correlation between TCGA-LGG *PODNL1* CpG methylations (beta-values) and *PODNL1* mRNA expressions (RSEM+1). Spearman's rho correlation coefficients are reported within each figure.

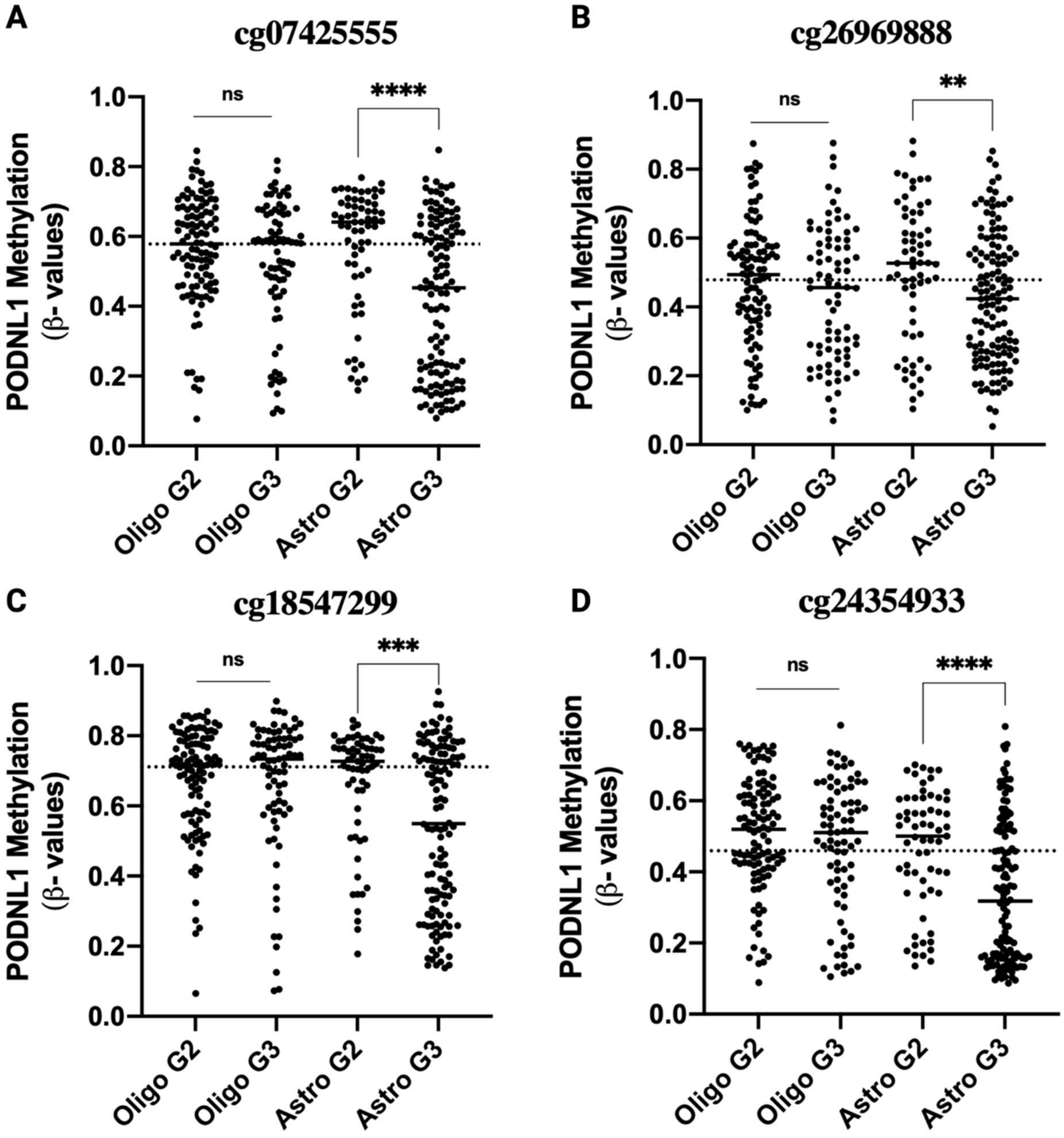

Figure S2. Association between PODNL1 CpG methylation levels and LGG histological subtypes and their tumor grades for (**A**) cg07425555 (**B**) cg26969888 (**C**) cg18547299, and (**D**) cg24354933. Dotted line represents median CpG methylation levels for LGG used for methylation status cutoff. **** $p < 0.0001$, *** $p < 0.001$, ** p < 0.01 and ns = not significant.

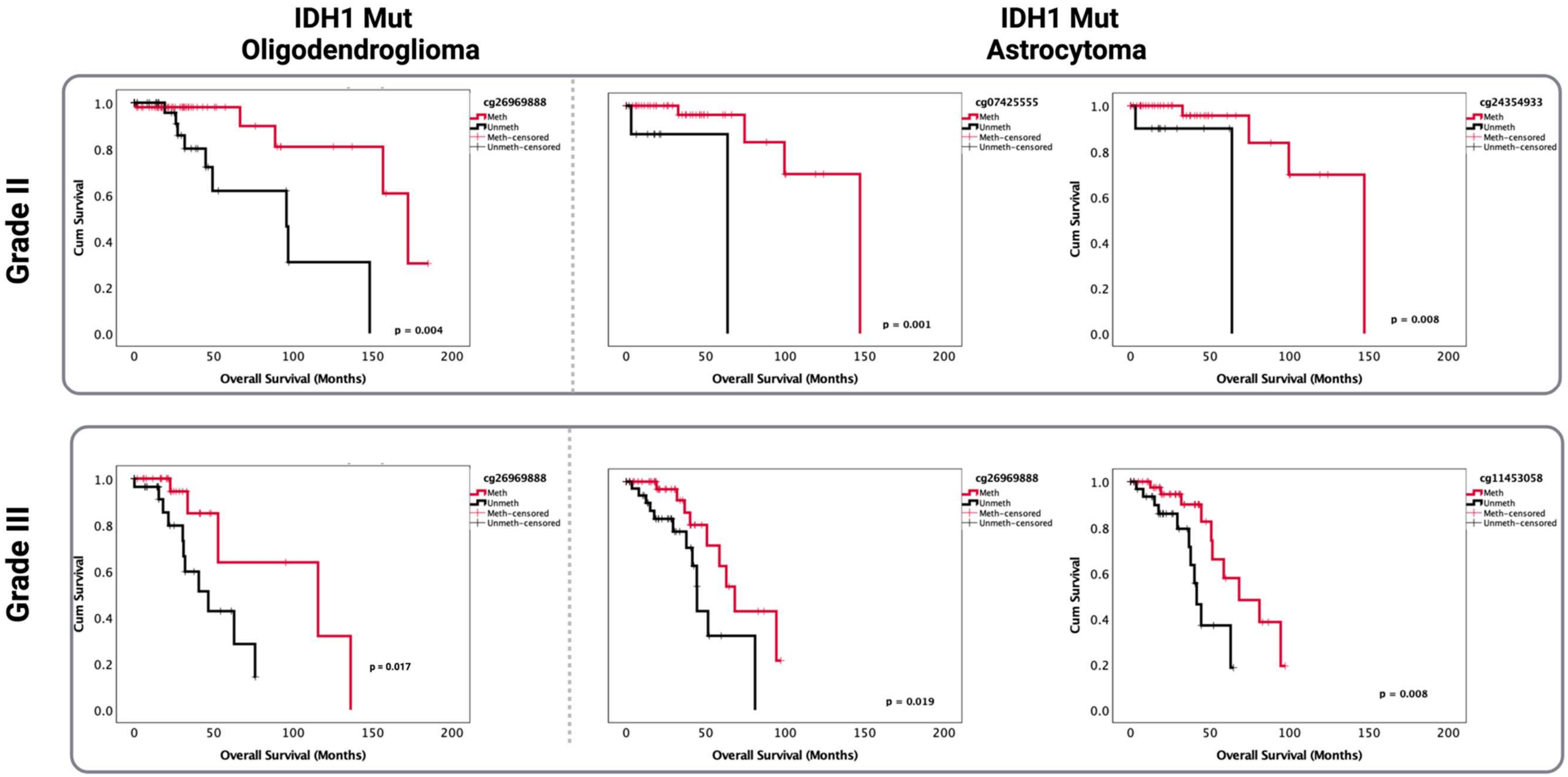

Figure S3. PODNL1 CpG site methylation status affects overall survival in grades II and II IDH1 mutant oligodendroglioma and astrocytoma. Red line indicates high methylation levels and Black line indicates low methylation levels, determined by median beta-value cutoff for each CpG. Log-rank *p*-values are marked in each figure. A *p*-value of < 0.05 was considered significant.

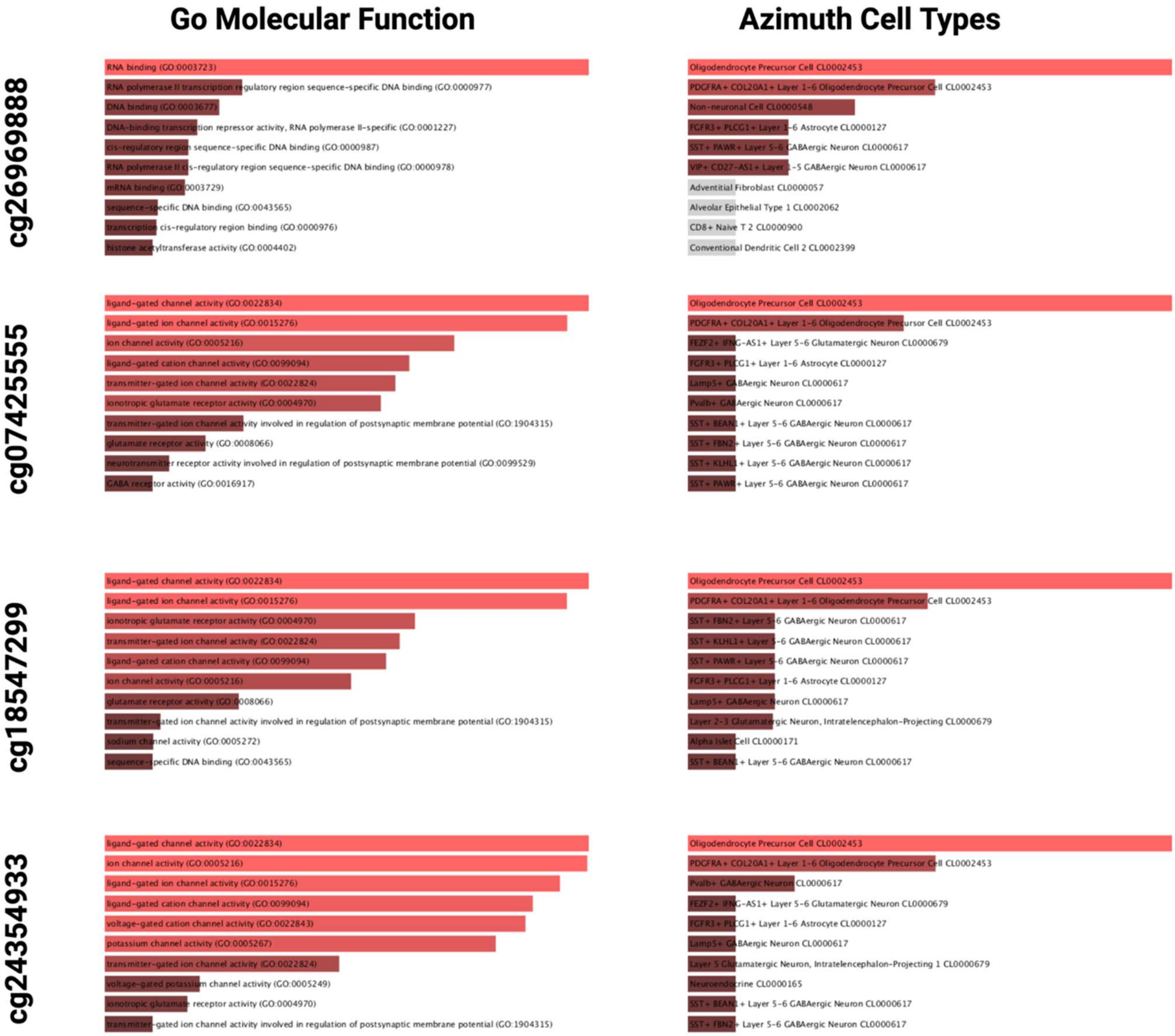

Figure S4. Gene Ontology (GO) molecular function and Azimuth cell type analysis using top 1000 significantly upregulated genes (false discovery rate < 0.01) in *PODNL1* high methylation groups of four significant CpGs. Data represents $-\log_{10}$ *p*-values. Enriched terms have *p*-values < 0.05, except grey bars where *p*-values were > 0.05.

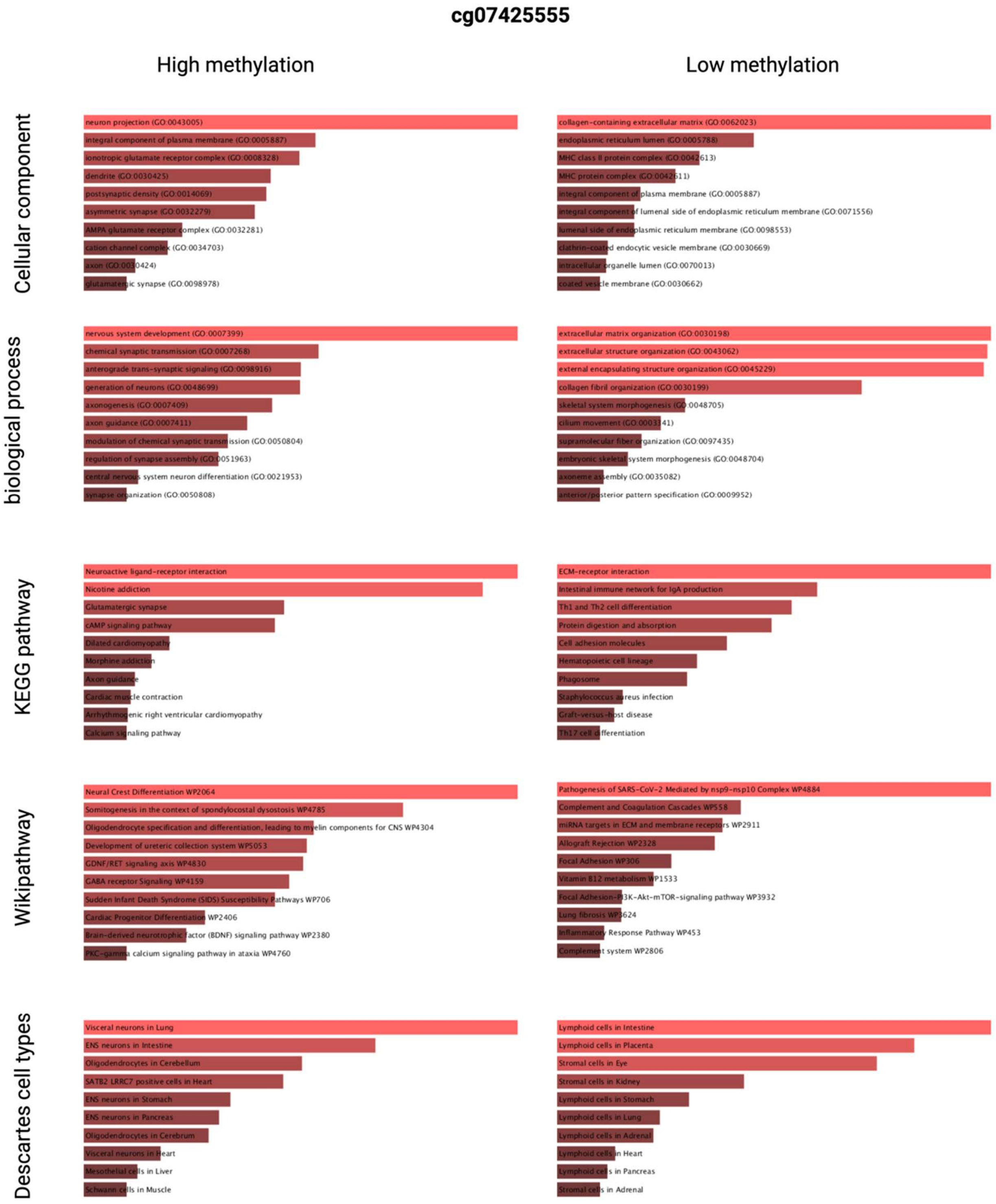

cg07425555
High methylation
Low methylation
Cellular component
neuron projection (GO:0043005)
integral component of plasma membrane (GO:0005887)
ionotropic glutamate receptor complex (GO:0008328)
dendrite (GO:0030425)
postsynaptic density (GO:0014069)
asymmetric synapse (GO:0032279)
AMPA glutamate receptor complex (GO:0032281)
cation channel complex (GO:0034703)
axon (GO:0030424)
glutamatergic synapse (GO:0098978)
collagen-containing extracellular matrix (GO:0062023)
endoplasmic reticulum lumen (GO:0005788)
MHC class II protein complex (GO:0042613)
MHC protein complex (GO:0042611)
integral component of plasma membrane (GO:0005887)
integral component of lumenal side of endoplasmic reticulum membrane (GO:0071556)
lumenal side of endoplasmic reticulum membrane (GO:0098553)
clathrin-coated endocytic vesicle membrane (GO:0030669)
intracellular organelle lumen (GO:0070013)
coated vesicle membrane (GO:0030662)
biological process
nervous system development (GO:0007399)
chemical synaptic transmission (GO:0007268)
anterograde trans-synaptic signaling (GO:0098916)
generation of neurons (GO:0048699)
axonogenesis (GO:0007409)
axon guidance (GO:0007411)
modulation of chemical synaptic transmission (GO:0050804)
regulation of synapse assembly (GO:0051963)
central nervous system neuron differentiation (GO:0021953)
synapse organization (GO:0050808)
extracellular matrix organization (GO:0030198)
extracellular structure organization (GO:0043062)
external encapsulating structure organization (GO:0045229)
collagen fibril organization (GO:0030199)
skeletal system morphogenesis (GO:0048705)
cilium movement (GO:0003341)
supramolecular fiber organization (GO:0097435)
embryonic skeletal system morphogenesis (GO:0048704)
axoneme assembly (GO:0035082)
anterior/posterior pattern specification (GO:0009952)
KEGG pathway
Neuroactive ligand-receptor interaction
Nicotine addiction
Glutamatergic synapse
cAMP signaling pathway
Dilated cardiomyopathy
Morphine addiction
Axon guidance
Cardiac muscle contraction
Arrhythmogenic right ventricular cardiomyopathy
Calcium signaling pathway
ECM-receptor interaction
Intestinal immune network for IgA production
Th1 and Th2 cell differentiation
Protein digestion and absorption
Cell adhesion molecules
Hematopoietic cell lineage
Phagosome
Staphylococcus aureus infection
Graft-versus-host disease
Th17 cell differentiation
Wikipathway
Neural Crest Differentiation WP2064
Somitogenesis in the context of spondylocostal dysostosis WP4785
Oligodendrocyte specification and differentiation, leading to myelin components for CNS WP4304
Development of ureteric collection system WP5053
GDNF/RET signaling axis WP4830
GABA receptor Signaling WP4159
Sudden Infant Death Syndrome (SIDS) Susceptibility Pathways WP706
Cardiac Progenitor Differentiation WP2406
Brain-derived neurotrophic factor (BDNF) signaling pathway WP2380
PKC-gamma calcium signaling pathway in ataxia WP4760
Pathogenesis of SARS-CoV-2 Mediated by nsp9-nsp10 Complex WP4884
Complement and Coagulation Cascades WP558
miRNA targets in ECM and membrane receptors WP2911
Allograft Rejection WP2328
Focal Adhesion WP306
Vitamin B12 metabolism WP1533
Focal Adhesion-PI3K-Akt-mTOR-signaling pathway WP3932
Lung fibrosis WP3624
Inflammatory Response Pathway WP453
Complement system WP2806
Descartes cell types
Visceral neurons in Lung
ENS neurons in Intestine
Oligodendrocytes in Cerebellum
SATB2 LRRC7 positive cells in Heart
ENS neurons in Stomach
ENS neurons in Pancreas
Oligodendrocytes in Cerebrum
Visceral neurons in Heart
Mesothelial cells in Liver
Schwann cells in Muscle
Lymphoid cells in Intestine
Lymphoid cells in Placenta
Stromal cells in Eye
Stromal cells in Kidney
Lymphoid cells in Stomach
Lymphoid cells in Lung
Lymphoid cells in Adrenal
Lymphoid cells in Heart
Lymphoid cells in Pancreas
Stromal cells in Adrenal

Continued…

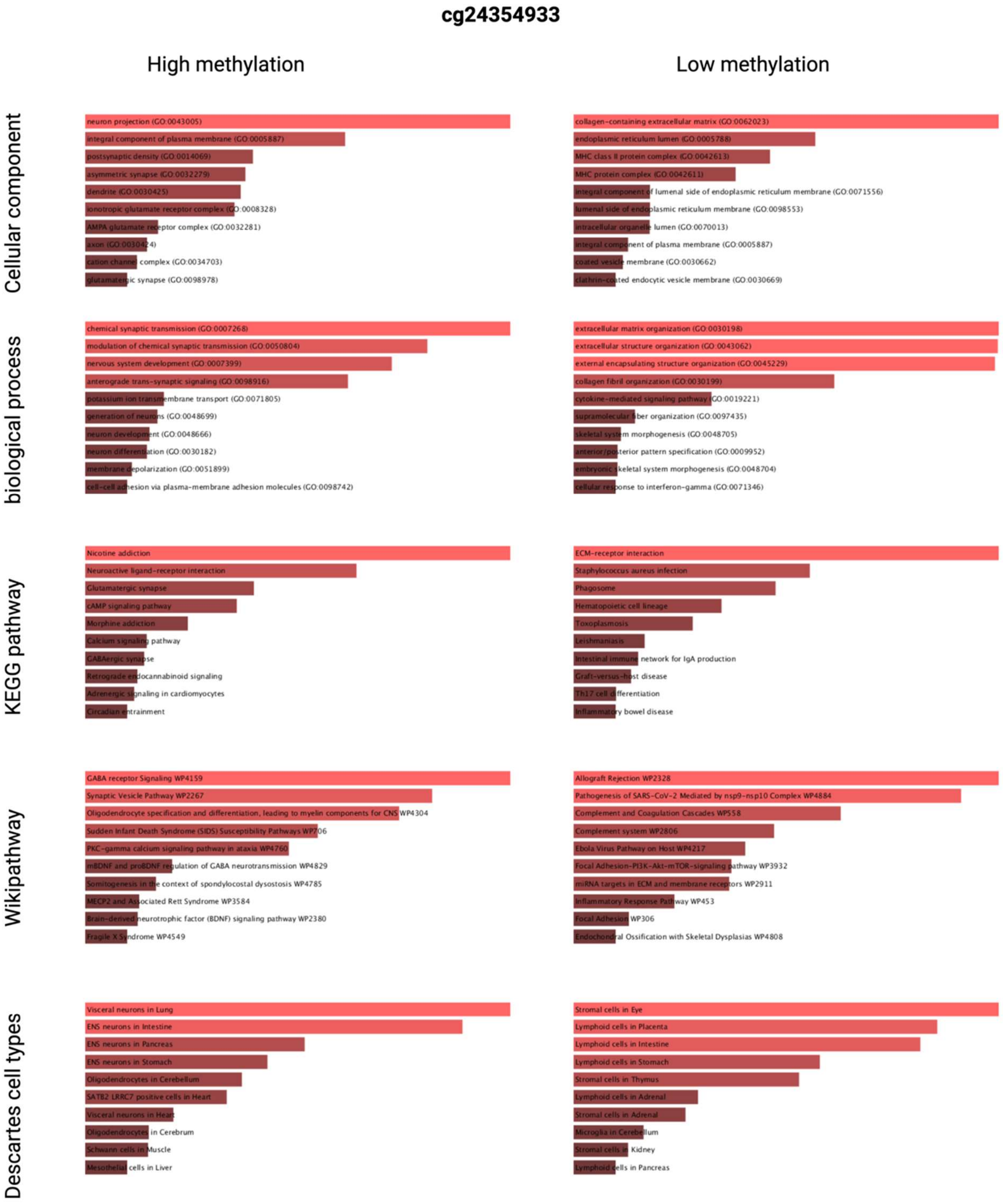
cg24354933
High methylation
Low methylation
Cellular component
neuron projection (GO:0043005)
integral component of plasma membrane (GO:0005887)
postsynaptic density (GO:0014069)
asymmetric synapse (GO:0032279)
dendrite (GO:0030425)
ionotropic glutamate receptor complex (GO:0008328)
AMPA glutamate receptor complex (GO:0032281)
axon (GO:0030424)
cation channel complex (GO:0034703)
glutamatergic synapse (GO:0098978)
collagen-containing extracellular matrix (GO:0062023)
endoplasmic reticulum lumen (GO:0005788)
MHC class II protein complex (GO:0042613)
MHC protein complex (GO:0042611)
integral component of lumenal side of endoplasmic reticulum membrane (GO:0071556)
lumenal side of endoplasmic reticulum membrane (GO:0098553)
intracellular organelle lumen (GO:0070013)
integral component of plasma membrane (GO:0005887)
coated vesicle membrane (GO:0030662)
clathrin-coated endocytic vesicle membrane (GO:0030669)
biological process
chemical synaptic transmission (GO:0007268)
modulation of chemical synaptic transmission (GO:0050804)
nervous system development (GO:0007399)
anterograde trans-synaptic signaling (GO:0098916)
potassium ion transmembrane transport (GO:0071805)
generation of neurons (GO:0048699)
neuron development (GO:0048666)
neuron differentiation (GO:0030182)
membrane depolarization (GO:0051899)
cell-cell adhesion via plasma-membrane adhesion molecules (GO:0098742)
extracellular matrix organization (GO:0030198)
extracellular structure organization (GO:0043062)
external encapsulating structure organization (GO:0045229)
collagen fibril organization (GO:0030199)
cytokine-mediated signaling pathway (GO:0019221)
supramolecular fiber organization (GO:0097435)
skeletal system morphogenesis (GO:0048705)
anterior/posterior pattern specification (GO:0009952)
embryonic skeletal system morphogenesis (GO:0048704)
cellular response to interferon-gamma (GO:0071346)
KEGG pathway
Nicotine addiction
Neuroactive ligand-receptor interaction
Glutamatergic synapse
cAMP signaling pathway
Morphine addiction
Calcium signaling pathway
GABAergic synapse
Retrograde endocannabinoid signaling
Adrenergic signaling in cardiomyocytes
Circadian entrainment
ECM-receptor interaction
Staphylococcus aureus infection
Phagosome
Hematopoietic cell lineage
Toxoplasmosis
Leishmaniasis
Intestinal immune network for IgA production
Graft-versus-host disease
Th17 cell differentiation
Inflammatory bowel disease
Wikipathway
GABA receptor Signaling WP4159
Synaptic Vesicle Pathway WP2267
Oligodendrocyte specification and differentiation, leading to myelin components for CNS WP4304
Sudden Infant Death Syndrome (SIDS) Susceptibility Pathways WP706
PKC-gamma calcium signaling pathway in ataxia WP4760
mBDNF and proBDNF regulation of GABA neurotransmission WP4829
Somitogenesis in the context of spondylocostal dysostosis WP4785
MECP2 and Associated Rett Syndrome WP3584
Brain-derived neurotrophic factor (BDNF) signaling pathway WP2380
Fragile X Syndrome WP4549
Allograft Rejection WP2328
Pathogenesis of SARS-CoV-2 Mediated by nsp9-nsp10 Complex WP4884
Complement and Coagulation Cascades WP558
Complement system WP2806
Ebola Virus Pathway on Host WP4217
Focal Adhesion-PI3K-Akt-mTOR-signaling pathway WP3932
miRNA targets in ECM and membrane receptors WP2911
Inflammatory Response Pathway WP453
Focal Adhesion WP306
Endochondral Ossification with Skeletal Dysplasias WP4808
Descartes cell types
Visceral neurons in Lung
ENS neurons in Intestine
ENS neurons in Pancreas
ENS neurons in Stomach
Oligodendrocytes in Cerebellum
SATB2 LRRC7 positive cells in Heart
Visceral neurons in Heart
Oligodendrocytes in Cerebrum
Schwann cells in Muscle
Mesothelial cells in Liver
Stromal cells in Eye
Lymphoid cells in Placenta
Lymphoid cells in Intestine
Lymphoid cells in Stomach
Stromal cells in Thymus
Lymphoid cells in Adrenal
Stromal cells in Adrenal
Microglia in Cerebellum
Stromal cells in Kidney
Lymphoid cells in Pancreas

Continued…

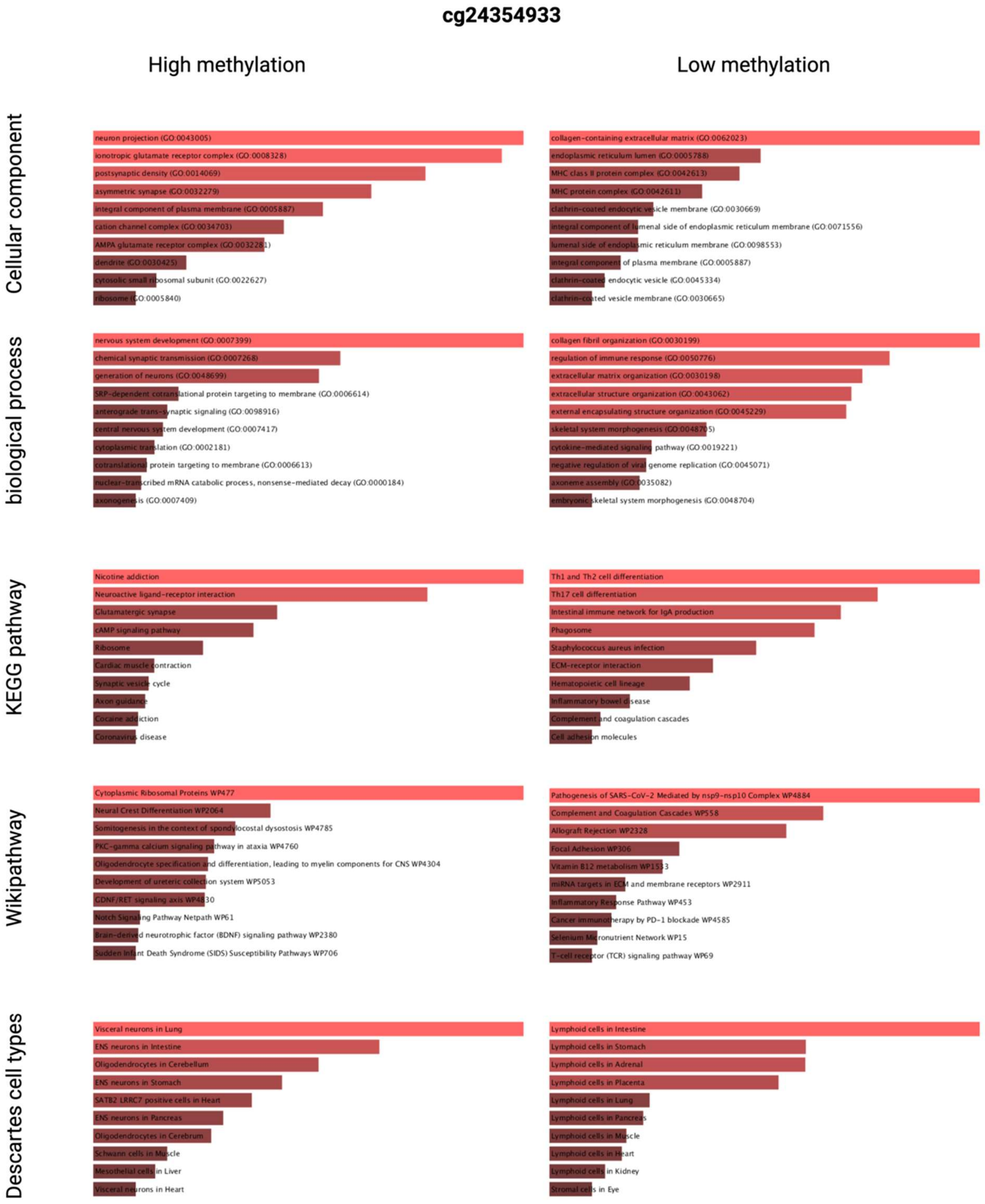
cg24354933
High methylation
Low methylation
Cellular component
neuron projection (GO:0043005)
ionotropic glutamate receptor complex (GO:0008328)
postsynaptic density (GO:0014069)
asymmetric synapse (GO:0032279)
integral component of plasma membrane (GO:0005887)
cation channel complex (GO:0034703)
AMPA glutamate receptor complex (GO:0032281)
dendrite (GO:0030425)
cytosolic small ribosomal subunit (GO:0022627)
ribosome (GO:0005840)
collagen-containing extracellular matrix (GO:0062023)
endoplasmic reticulum lumen (GO:0005788)
MHC class II protein complex (GO:0042613)
MHC protein complex (GO:0042611)
clathrin-coated endocytic vesicle membrane (GO:0030669)
integral component of lumenal side of endoplasmic reticulum membrane (GO:0071556)
lumenal side of endoplasmic reticulum membrane (GO:0098553)
integral component of plasma membrane (GO:0005887)
clathrin-coated endocytic vesicle (GO:0045334)
clathrin-coated vesicle membrane (GO:0030665)
biological process
nervous system development (GO:0007399)
chemical synaptic transmission (GO:0007268)
generation of neurons (GO:0048699)
SRP-dependent cotranslational protein targeting to membrane (GO:0006614)
anterograde trans-synaptic signaling (GO:0098916)
central nervous system development (GO:0007417)
cytoplasmic translation (GO:0002181)
cotranslational protein targeting to membrane (GO:0006613)
nuclear-transcribed mRNA catabolic process, nonsense-mediated decay (GO:0000184)
axonogenesis (GO:0007409)
collagen fibril organization (GO:0030199)
regulation of immune response (GO:0050776)
extracellular matrix organization (GO:0030198)
extracellular structure organization (GO:0043062)
external encapsulating structure organization (GO:0045229)
skeletal system morphogenesis (GO:0048705)
cytokine-mediated signaling pathway (GO:0019221)
negative regulation of viral genome replication (GO:0045071)
axoneme assembly (GO:0035082)
embryonic skeletal system morphogenesis (GO:0048704)
KEGG pathway
Nicotine addiction
Neuroactive ligand-receptor interaction
Glutamatergic synapse
cAMP signaling pathway
Ribosome
Cardiac muscle contraction
Synaptic vesicle cycle
Axon guidance
Cocaine addiction
Coronavirus disease
Th1 and Th2 cell differentiation
Th17 cell differentiation
Intestinal immune network for IgA production
Phagosome
Staphylococcus aureus infection
ECM-receptor interaction
Hematopoietic cell lineage
Inflammatory bowel disease
Complement and coagulation cascades
Cell adhesion molecules
Wikipathway
Cytoplasmic Ribosomal Proteins WP477
Neural Crest Differentiation WP2064
Somitogenesis in the context of spondylocostal dysostosis WP4785
PKC-gamma calcium signaling pathway in ataxia WP4760
Oligodendrocyte specification and differentiation, leading to myelin components for CNS WP4304
Development of ureteric collection system WP5053
GDNF/RET signaling axis WP4830
Notch Signaling Pathway Netpath WP61
Brain-derived neurotrophic factor (BDNF) signaling pathway WP2380
Sudden Infant Death Syndrome (SIDS) Susceptibility Pathways WP706
Pathogenesis of SARS-CoV-2 Mediated by nsp9-nsp10 Complex WP4884
Complement and Coagulation Cascades WP558
Allograft Rejection WP2328
Focal Adhesion WP306
Vitamin B12 metabolism WP1533
miRNA targets in ECM and membrane receptors WP2911
Inflammatory Response Pathway WP453
Cancer immunotherapy by PD-1 blockade WP4585
Selenium Micronutrient Network WP15
T-cell receptor (TCR) signaling pathway WP69
Descartes cell types
Visceral neurons in Lung
ENS neurons in Intestine
Oligodendrocytes in Cerebellum
ENS neurons in Stomach
SATB2 LRRC7 positive cells in Heart
ENS neurons in Pancreas
Oligodendrocytes in Cerebrum
Schwann cells in Muscle
Mesothelial cells in Liver
Visceral neurons in Heart
Lymphoid cells in Intestine
Lymphoid cells in Stomach
Lymphoid cells in Adrenal
Lymphoid cells in Placenta
Lymphoid cells in Lung
Lymphoid cells in Pancreas
Lymphoid cells in Muscle
Lymphoid cells in Heart
Lymphoid cells in Kidney
Stromal cells in Eye

Continued…

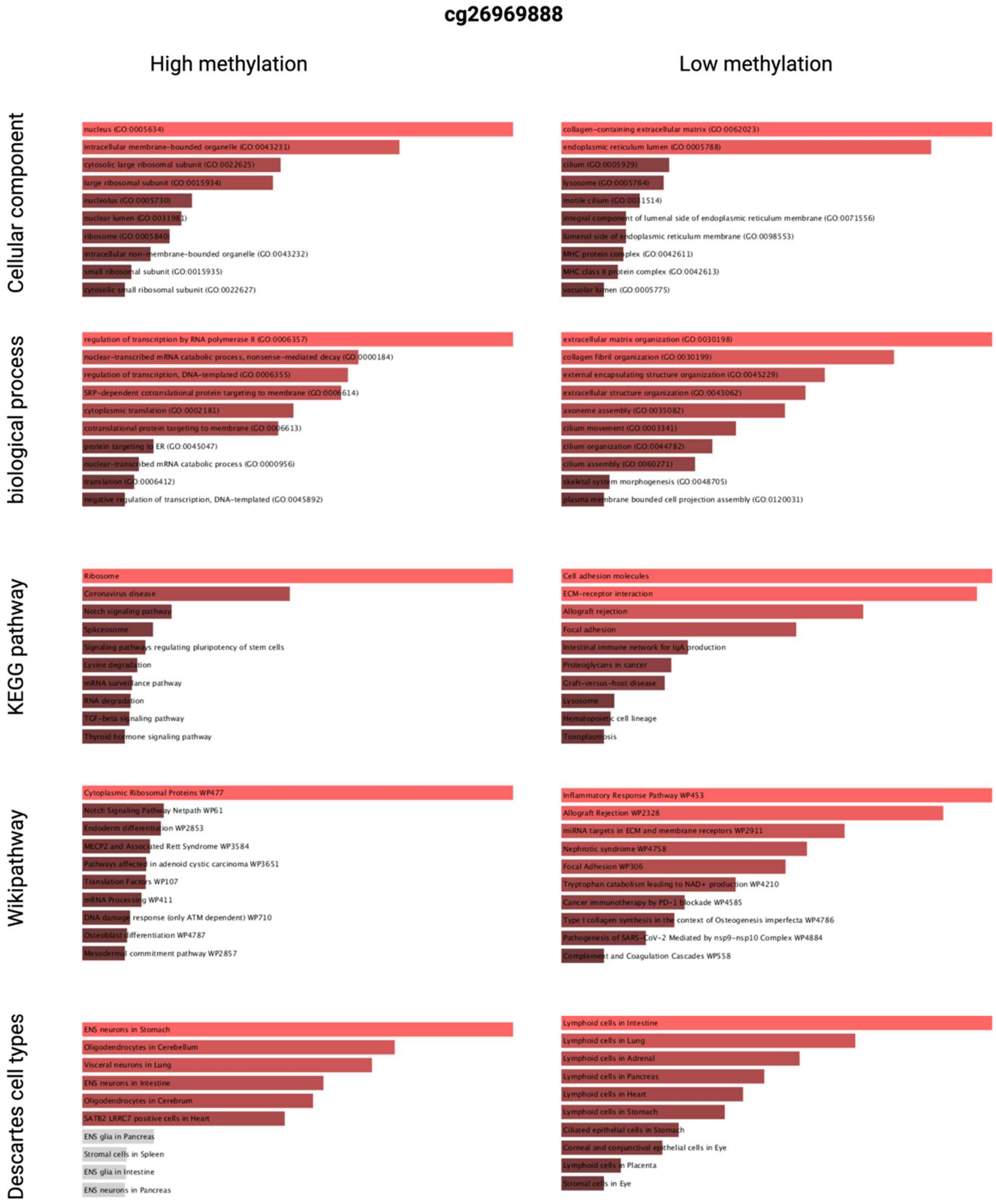

Figure S5. Gene Ontology (GO) Cellular Component, Biological Process, KEGG pathway, WikiPathway and Descartes cell type analysis using top 1000 significantly upregulated genes (false discovery rate < 0.01) in *PODNL1* high and low methylation groups of four significant CpGs. Data represents -$\log_{10}$ p-values. Enriched terms have *p*-values < 0.05, except grey bars where *p*-values were > 0.05.

cg24354933

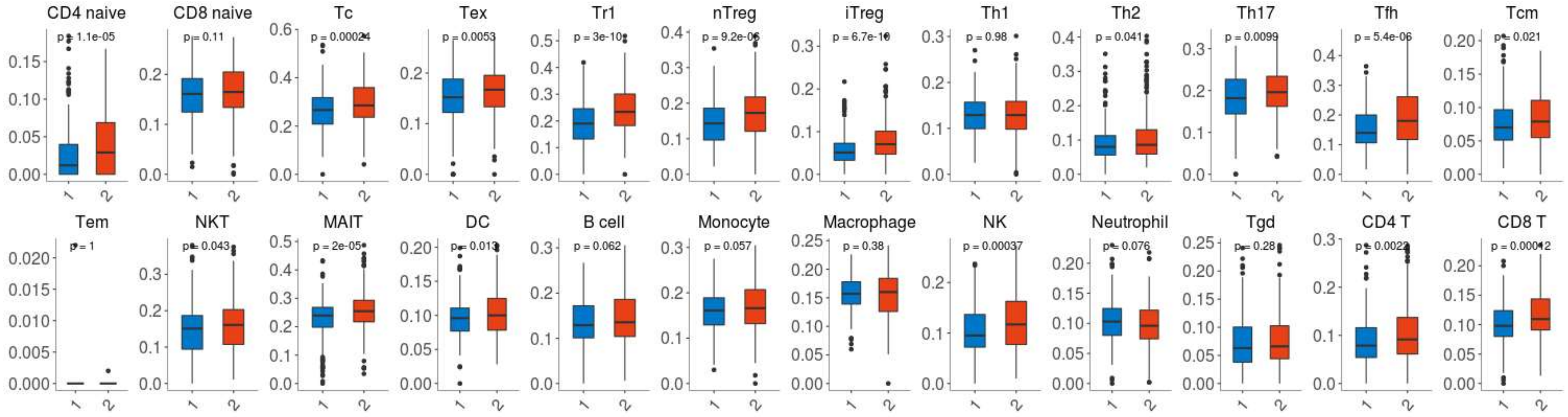

cg26969888

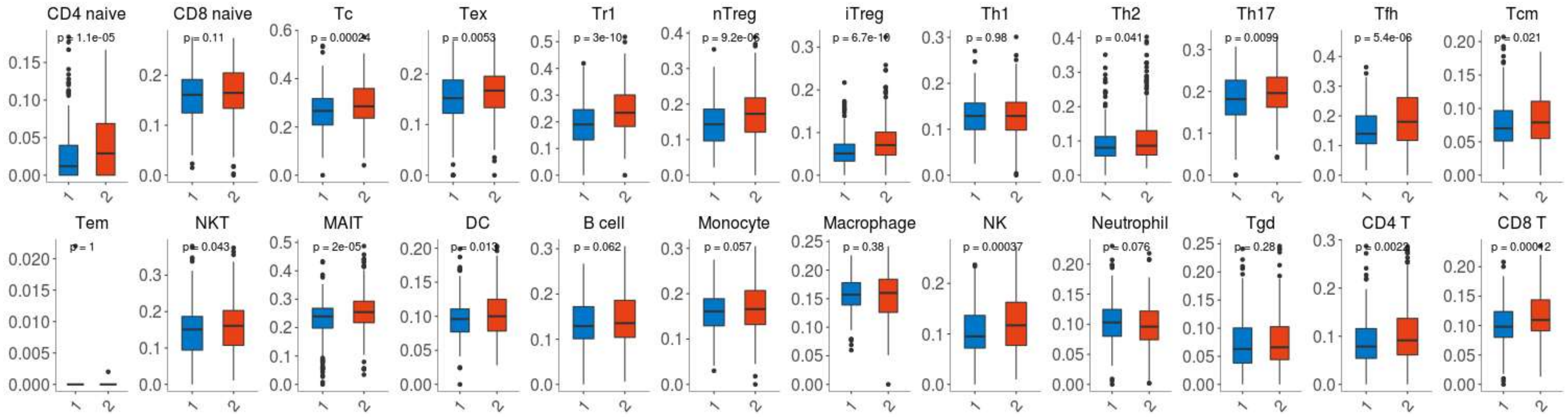

Continued…

cg18547299

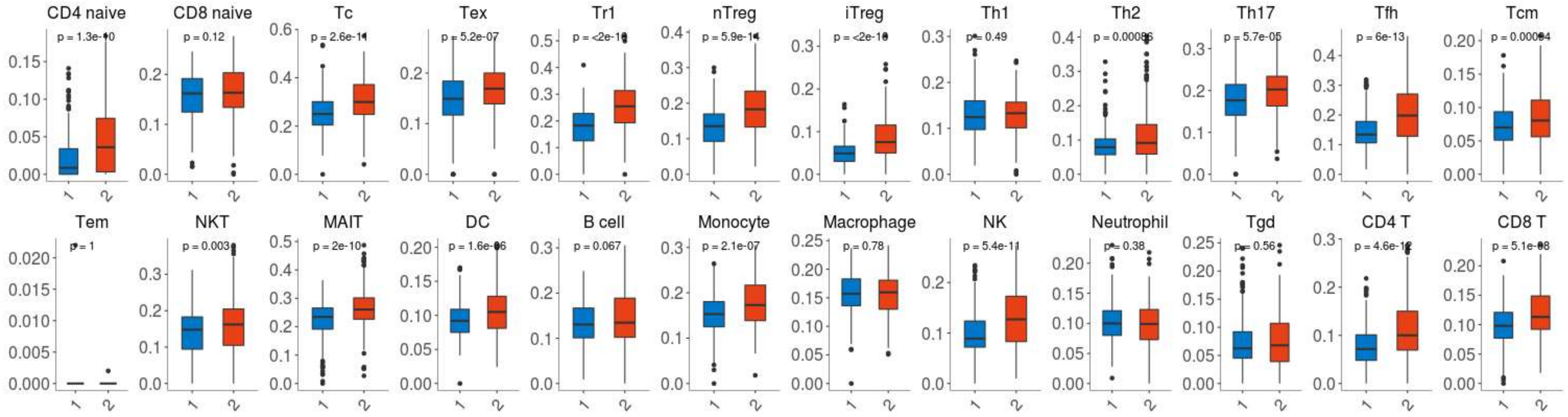

cg07425555

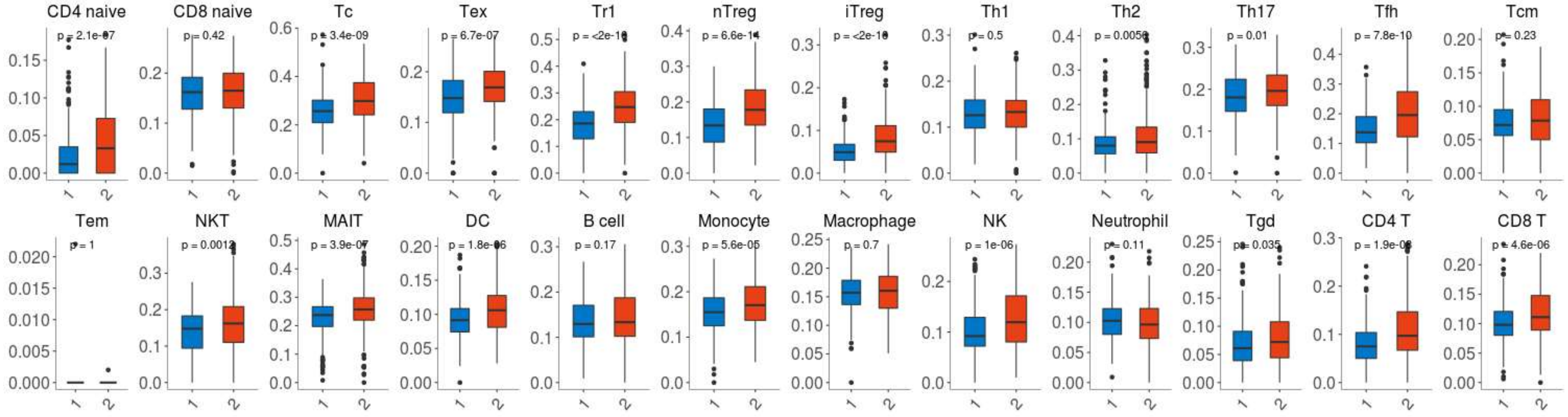

Figure S6. Association between specific PODNL1 CpG methylation group (group 1= high methylation and group 2 = low methylation; stratified by median beta value cut-off) and infiltrating immune cells in TCGA LGG for all four significant CpGs. Statistical significance was determined by *t*-test with a *p*-value < 0.05.

OLIGODENDROGLIOMA

cg18547299

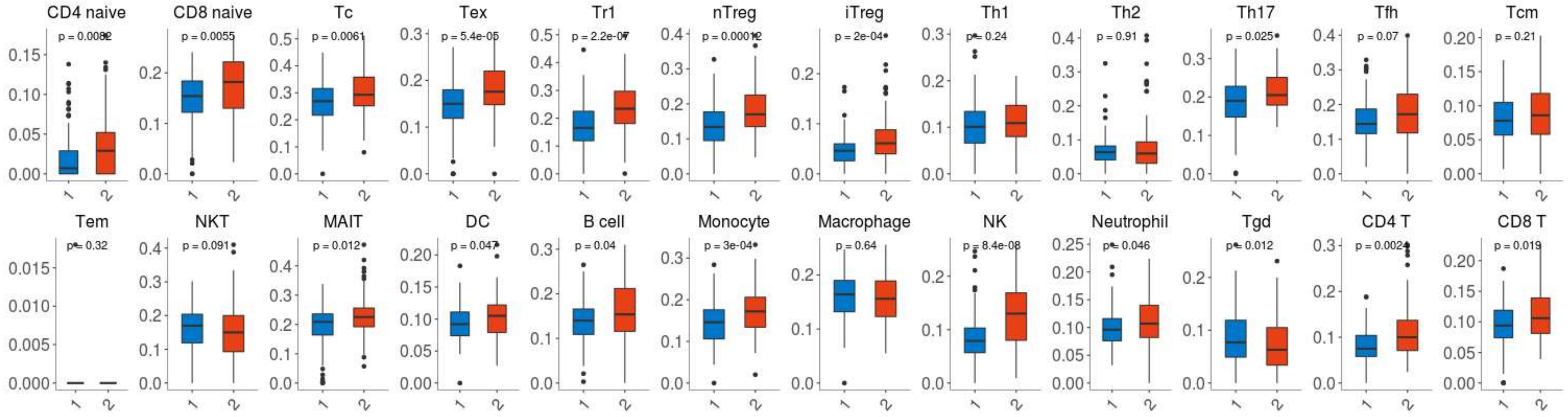

cg07425555

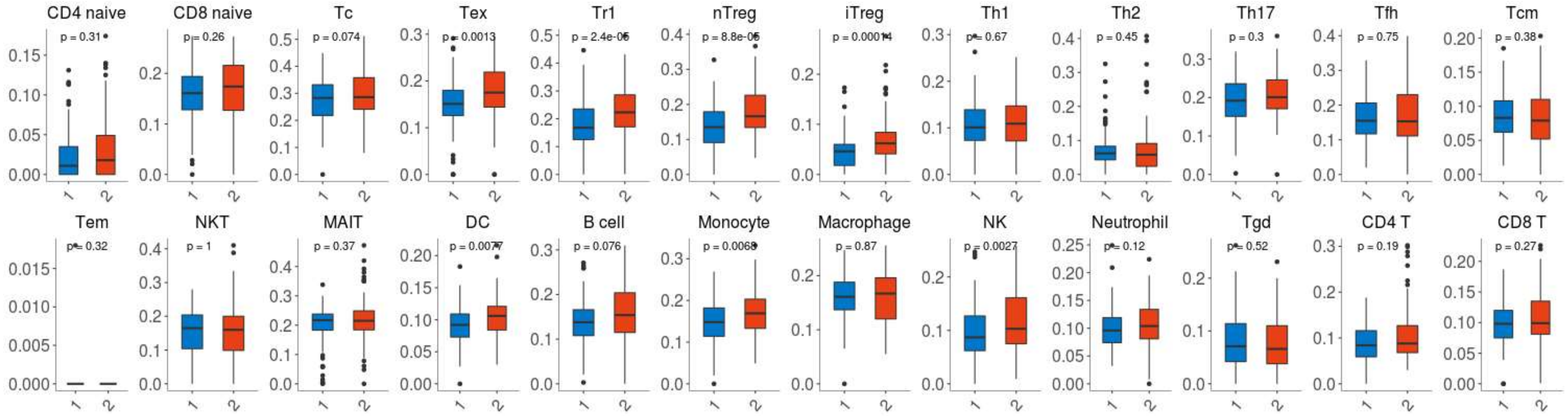

Continued…

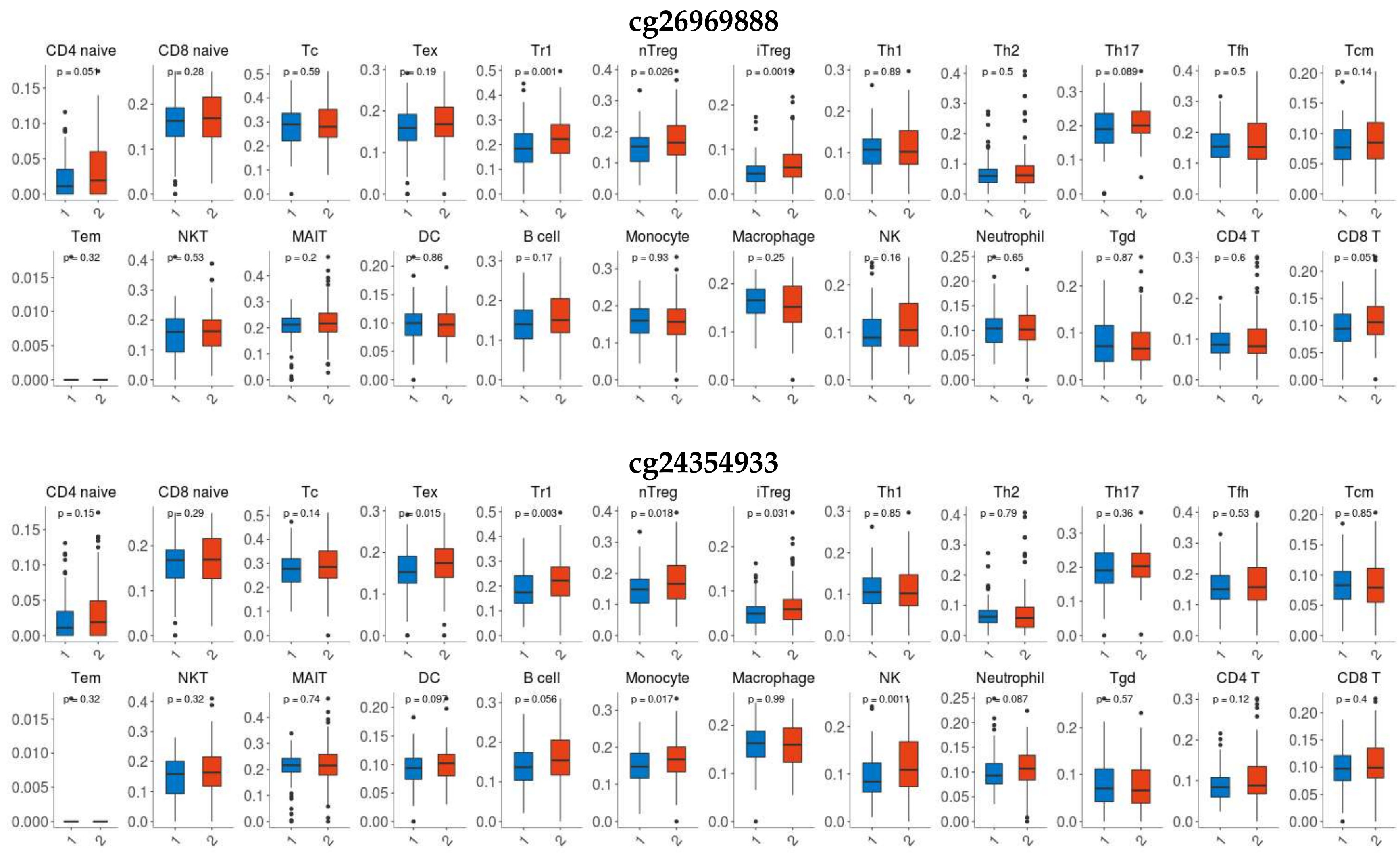

Figure S7. Association between specific PODNL1 CpG methylation group (group 1= high methylation and group 2 = low methylation; stratified by median beta value cut-off) and infiltrating immune cells in TCGA Oligodendroglioma for all four significant CpGs. Statistical significance was determined by *t*-test with a *p*-value < 0.05.

ASTROCYTOMA

cg18547299

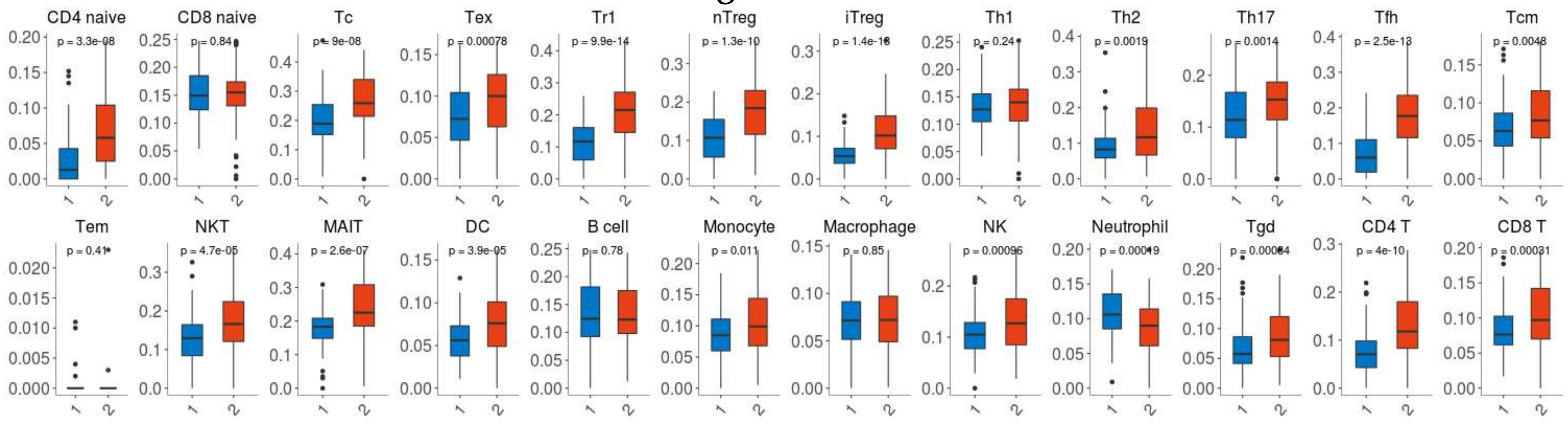

cg07425555

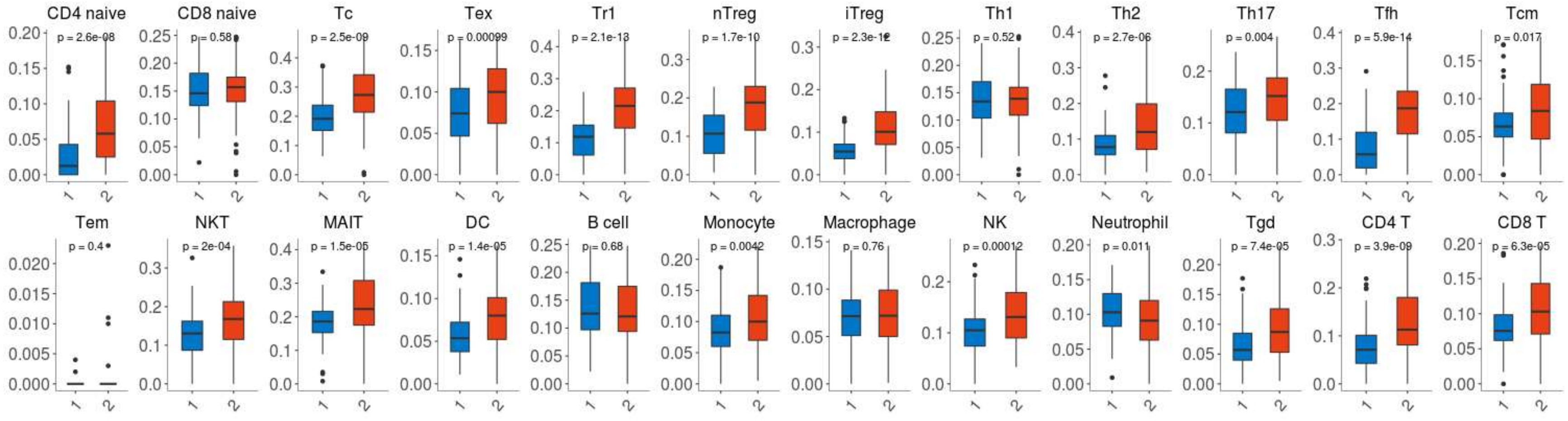

Continued…

cg26969888

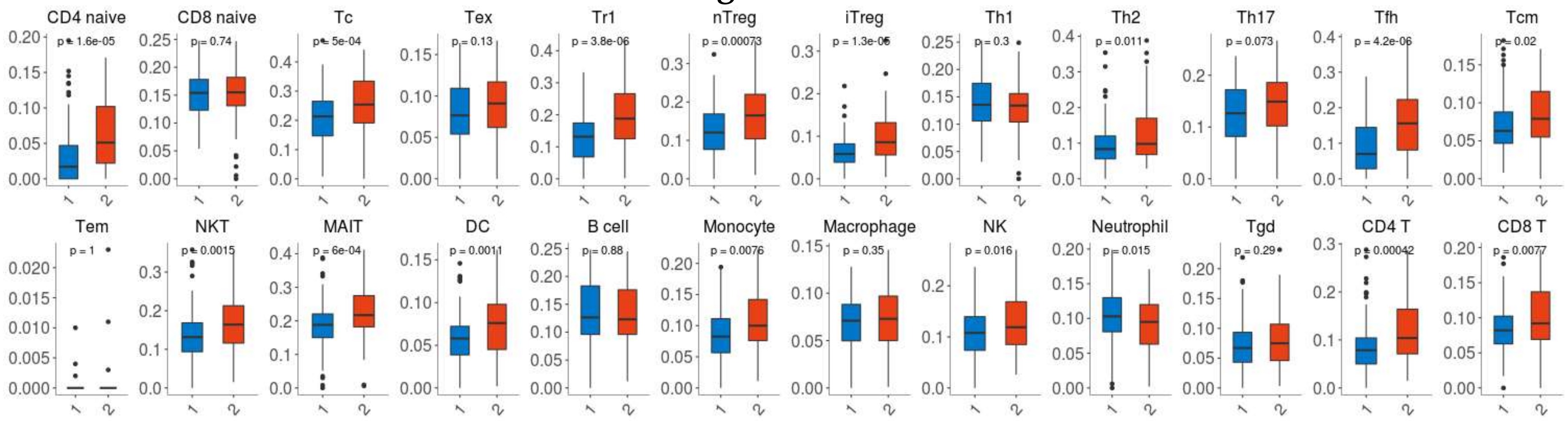

cg24354933

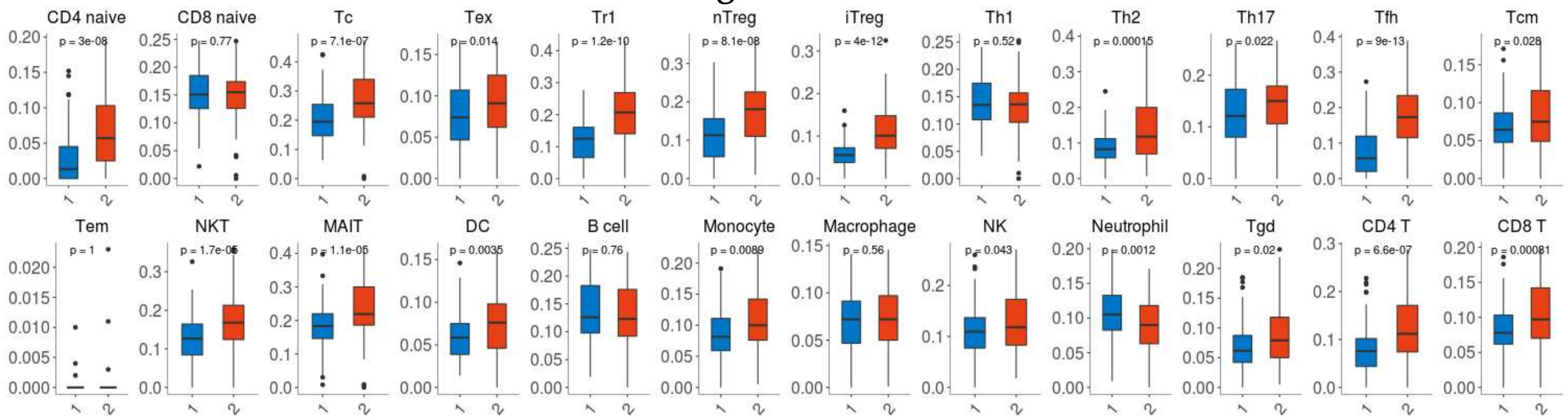

Figure S8. Association between specific PODNL1 CpG methylation group (group 1= high methylation and group 2 = low methylation; stratified by median beta value cut-off) and infiltrating immune cells in TCGA Astrocytoma for all four significant CpGs. Statistical significance was determined by *t*-test with a *p*-value < 0.05
